# Transmissible α-synuclein seeding activity in brain and stomach of patients with Parkinson’s disease

**DOI:** 10.1007/s00401-021-02312-4

**Published:** 2021-04-24

**Authors:** Achim Thomzig, Katja Wagenführ, Phillip Pinder, Marion Joncic, Walter J. Schulz-Schaeffer, Michael Beekes

**Affiliations:** 1 Prion and Prionoid Research Unit, ZBS 6—Proteomics and Spectroscopy, ZBS—Centre for Biological Threats and Special Pathogens, Robert Koch Institute, Nordufer 20, 13353 Berlin, Germany; 2 Present Address: State Office for Health and Social Affairs (LAGeSo), Berlin, Germany; 3 Institute of Neuropathology, Faculty of Medicine, Saarland University, Homburg, Germany

**Keywords:** Parkinson´s disease, Alpha-synuclein, Seeding, Transmission, Mouse bioassay, Stomach

## Abstract

Cerebral deposition of abnormally aggregated α-synuclein (αSyn) is a neuropathological hallmark of Parkinson’s disease (PD). PD-associated αSyn (αSyn^PD^) aggregates can act as proteinaceous nuclei (“seeds”) able of self-templated propagation. Since this is strikingly reminiscent to properties of proteinaceous infectious particles (prions), lessons learned from prion diseases suggest to test whether transferred αSyn^PD^ can propagate and induce neurological impairments or disease in a new host. Two studies that addressed this question provided divergent results. Intracerebral (i.c.) injection of Lewy body extracts from PD patients caused cerebral αSyn pathology, as well as nigrostriatal neurodegeneration, of wild-type mice and macaques, with the mice also showing motor impairments (Recasens et al. 2014, Ann Neurol 75:351–362). In contrast, i.c. transmission of homogenates from PD brains did not stimulate, after “> 360” days post-injection (dpi), pathological αSyn conversion or clinical symptoms in transgenic TgM83^+/−^ mice hemizygously expressing mutated (A53T) human αSyn (Prusiner et al. 2015, PNAS 112:E5308–E5317). To advance the assessment of possible αSyn^PD^ hazards by providing further data, we examined neuropathological and clinical effects upon i.c. transmission of brain, stomach wall and muscle tissue as well as blood from PD patients in TgM83^+/−^ mice up to 612 dpi. This revealed a subtle, yet distinctive stimulation of localized αSyn aggregation in the somatodendritic compartment and dystrophic neurites of individual or focally clustered cerebral neurons after challenge with brain and stomach wall homogenates. No such effect was observed with transmitted blood or homogenized muscle tissue. The detected stimulation of αSyn aggregation was not accompanied by apparent motor impairments or overt neurological disease in TgM83^+/−^ mice. Our study substantiated that transmitted αSyn^PD^ seeds, including those from the stomach wall, are able to propagate in new mammalian hosts. The consequences of such propagation and potential safeguards need to be further investigated.

**Supplementary Information:**

The online version contains supplementary material available at 10.1007/s00401-021-02312-4.

## Introduction

Numerous findings indicate that pathologically aggregated species of amyloid-β (Aβ) and tau protein in Alzheimer’s disease (AD), and of α-synuclein (αSyn) in Parkinson’s disease (PD) can act as proteinaceous nuclei (‘seeds’) which recruit endogenous precursor proteins and convert them into their own misfolded oligomeric or polymeric aggregate structures. Such self-templated propagation of pathological protein states in AD and PD shows obvious parallels to the replication of proteinaceous infectious particles (prions) in transmissible spongiform encephalopathies that essentially consist of pathologically misfolded and aggregated prion protein (reviewed in: [7, 19, 24, 38]). This has raised the question of whether neuropathological or clinical features of AD or PD can be transmitted, in a prion-like way, between individuals by self-propagating protein seeds potentially present on medical devices such as surgical instruments, or associated with vehicles such tissue transplants, blood or blood products (reviewed or commented on in: [1, 6–8, 15, 19]).

Recent evidence suggests that possibly harmful transmissions of Aβ seeds of AD or cerebral amyloid angiopathy (CAA) did occur in the past between humans via contaminated human cadaveric pituitary growth hormone or dura mater transplants [3, 20, 21, 30]. In addition, further findings highlight the possibility that cerebral Aβ pathology and CAA may be transmissible, similarly to prion diseases, through neurosurgery [20]. This reinforces the question whether iatrogenic transmission risks also exist for αSyn seeds of PD, the second most common and fastest growing neurodegenerative disease in the world.

So far, there are no reports on the transmission of αSyn aggregation pathology or clinical αSyn aggregation disease from human to human. However, when the effects of an intracerebral (i.c.) transfer of test materials containing αSyn^PD^ in Lewy body (LB) extracts or homogenized brain tissue from PD patients were examined in two animal studies this produced contrasting findings. Recasens et al. [31] reported that in wild-type (wt) mice and macaques, stereotactical injection of human LB extracts into the substantia nigra or striatum caused a pathological conversion, accumulation, and phosphorylation of endogenous αSyn in different brain areas, as well as progressive nigrostriatal neurodegeneration. Furthermore, mice exposed as described to LB extracts from PD patients showed impaired motor coordination when examined in the pole test. In contrast, brain tissue homogenates prepared from PD patients that contained αSyn^PD^ were found by Prusiner et al. [29] to not stimulate pathological cerebral αSyn conversion or clinical symptoms after “> 360” dpi upon i.c. injection into transgenic TgM83^+/−^ mice hemizygously expressing human αSyn with the A53T mutation of familial PD.

The contrasting results of the two studies are not straightforward to explain and substantially complicate risk assessments that are needed to deal appropriately with the unclear transmission risks of αSyn^PD^ seeds. To gain further clarity, we felt that transmission experiments in TgM83^+/−^ mice should be deepened with incubation periods as long as possible and sample materials preferably containing higher amounts of αSyn^PD^ seeds than examined by Prusiner et al. [29]. Several different studies have demonstrated that neuritic αSyn pathology is much more abundant than perikaryal inclusions in different αSyn aggregation diseases [13, 14, 16], and up to 90% or even more of pathological cerebral αSyn aggregates were found being not localized in LBs but at presynapses in the form of much smaller micro-deposits [33, 34]. Therefore, when selecting the cerebral PD tissue to be tested we focused our attention specifically on brain regions with high levels of presynaptic αSyn^PD^ micro-aggregates and pronounced αSyn seeding activity, rather than on those with numerous LBs. Since PD patients exhibit substantial amounts of pathological αSyn^PD^ micro-aggregates at synapses in the caudate nucleus (W.J. Schulz-Schaeffer, pers. communication), we prepared cerebral test samples for i.c. injection into TgM83^+/−^ mice from this brain area.

In addition, samples of stomach wall tissue, skeletal muscle (biceps) and whole blood from a PD patient were tested in the TgM83^+/−^ bioassay. Stomach wall components such as ganglia of the enteric nervous system (ENS), but not skeletal muscle or blood, have been demonstrated to contain pathological αSyn^PD^ aggregates in PD patients (reviewed in: [23]). The bioassay testing of these sample materials in TgM83^+/−^ mice was intended to provide further information about the presence and transmission properties of αSyn^PD^ seeds in non-central nervous system tissues of PD patients.

## Materials and methods

### Human tissues

The work described in this report has been carried out in accordance with The Code of Ethics of the World Medical Association (Declaration of Helsinki) for experiments involving humans. The sampling and use of tissues from PD patients and human donors without PD for scientific purposes were undertaken with the understanding and written consent of each donor or his or her authorized caregiver. The use of the tissues for experimental studies and scientific reports such as our work was approved by the Ethics Committees of the University Medical Center Göttingen (No. 11/11/93).

The used tissues originated from four deceased donors, two of which had been clinically and neuropathologically diagnosed with PD (referred to as PD patients A [male, 87 years old] and B [male, 72 years old]). The donor of negative control brain tissue was not affected by PD or any other cerebral protein aggregation disease (referred to as control donor 1 [female, 28 years old]), while the donor of negative control stomach wall tissue was a patient deceased from or with Covid-19 without a medical history of PD (referred to as control donor 2 [male, 61 years old]). The brain tissue specimens of the PD patients and of control donor 1 were dissected from the caudate nucleus. Furthermore, tissue specimens from skeletal muscle (biceps) and the stomach wall, as well as whole blood without anticoagulants, were sampled from PD patient A. Negative control stomach wall tissue was taken from control donor 2. 10% (w/v) homogenates of PD and negative control brain tissue were prepared in sterile phosphate-buffered saline (PBS; 137 mM NaCl, 2.7 mM KCl, 6.5 mM Na_2_HPO_4_, 1.5 mM KH_2_PO_4_, adjusted to pH 7.4) by sonication. Muscle and stomach wall tissue from PD patient A was minced for preparation of 10% (w/v) homogenates in PBS (pH 7.4; 10% [w/v]) using an Ultra-Turrax device (IKA Werke, Staufen, Germany) and subsequently sonicated.

For in vitro analysis of αSyn^PD^ seeding activity by real-time quaking-induced conversion (RT-QuIC) assay 10% (w/v) stomach wall homogenates from PD patient A and control donor 2 were prepared in PBS using a Minilys Homogenisator (Bertin Instruments, Frankfurt, Germany) and sonication. However, since control donor 2 had Covid-19, coronavirus SARS-CoV-2 had to be inactivated reliably in stomach wall samples from this donor. According to current data on the heat inactivation of SARS-CoV-2 [4, 5] and an additional margin of safety, we, therefore, heated the stomach wall tissue from control donor 2 in a water bath to 65 °C for 45 min. in parafilm-sealed sample vessels prior to homogenization. The same was done for RT-QuIC assays with a subset of stomach wall specimens from PD patient A to match tissue-processing conditions.

Aliquots of tissue homogenates and whole blood were stored at − 80 °C until use. Homogenates of PD and non-PD brain tissues are referred to in this report as PBH (PD brain homogenate) and NBH (non-PD brain homogenate), respectively, with PBH A and PBH B designating PBH from PD patients A or B. Homogenates of muscle and stomach wall as well as whole blood from PD patient A are referred to as PMuH (PD muscle homogenate), PStH (PD stomach homogenate) and PBld (PD blood), respectively, while non-PD homogenate of stomach wall tissue from control donor 2 is referred to as NStH (non-PD stomach homogenate).

### Use of animals

All animal experiments were conducted under the European directive regarding the protection of animals used for experimental and other scientific purposes in strict accordance with the German Animal Welfare Act (Tierschutzgesetz) and adhering to the guidelines for the practical implementation of the German Animal Welfare Act published by the Charité—University Medicine Berlin. The protocol for animal experimentation was reviewed and approved by the responsible Committee on the Ethics of Animal Experiments (“Tierversuchskommission—Berlin”) affiliated at the Authority for Animal Protection in Berlin (“Landesamt für Gesundheit und Soziales Berlin”, Berlin, Germany; Permit Number G 0288/13). All surgery was performed under Ketavet/Xylazine anesthesia, and all efforts were made to minimize any suffering of animals. Euthanasia of wild-type mice for the preparation of brain tissue (which not required an approval for animal experiments) was reported to and registered by the animal protection authority (“Landesamt für Gesundheit und Soziales Berlin”, Berlin, Germany; Registration Number T0197/15). Homogenization of mouse brain tissue samples was performed in sterile PBS (pH 7.4) by sonication. Aliquots of homogenates were stored at − 80 °C until use.

### Biometrical study planning

The initial aim of the study was to elucidate by bioassay in TgM83^+/−^ mice whether brain tissue homogenates from the two PD patients A and B could be classified as free of αSyn^PD^ seeding activity according to a given prevalence threshold (which specified the maximum tolerable proportion of positive specimens in the sample population), or whether αSyn^PD^ seeding activity could be detected with statistical significance in the examined sample materials. In an attempt to increase the sensitivity of our bioassay as compared to a previous study by Prusiner et al. [29] animals should be kept, according to our study schedule, for incubation periods of at least 510 and up to 612 dpi before neuropathological examination. However, all mice having reached an incubation period of ≥ 400 dpi in the event of unscheduled euthanasia (e.g., due to interfering health problems not related to the purpose of this study) or spontaneous death were also included in the analysis.

20 μl aliquots of 10% (w/v) brain homogenates containing 2 mg of homogenized caudate nucleus tissue from PD patients A and B were used as intracerebral (i.c.) inoculation samples, with the prevalence threshold set to 10% and the probability for a type I error set to 5% (i.e., significance level *α* = 0.05). With these parameters, the required minimum animal number per test group was 28, based on a calculation according to Glaser and Kreienbrock [17]. This figure was also obtained by applying a simplified method for estimating the minimum group size according to Dell et al. [12] and the National Research Council (US) Committee on Guidelines for the Use of Animals in Neuroscience and Behavioral Research [25]. Therefore, including a safety reserve of 2 animals, 30 animals per group (15 females and males each) were planned to be inoculated each with brain homogenate from PD patients A and B. In the case of a negative test result (i.e., when in a group of at least 28 recipient animals none showed indications for exogenously stimulated αSyn aggregation), it could be concluded with a 90% confidence level that in the sample not more than half of the sample aliquots contained one seeding dose. Since this would be biologically equivalent to one 50% seeding dose (SD_50_) in TgM83^+/−^ mice per sample aliquot, a negative test result would indicate, with a 90% confidence level, that the tested brain tissue contained at most 500 SD_50_ per gram in these indicator mice.

As control group for the specificity of the bioassay, 40 TgM83^+/−^ mice (20 females and males each) were similarly i.c. challenged with 10% (w/v) brain homogenate from control donor 1 not affected by PD or any other cerebral protein aggregation disease.

In case that αSyn^PD^ seeding activity in brain tissue could be consistently detected, we had planned to subsequently also test one blood, muscle and stomach wall sample each from the patient with the most positive brain tissue result. However, given the already successful detection of αSyn^PD^ seeding activity in that case, it seemed appropriate to reduce the group size of the bioassay animals according to the principles of the 3Rs [18] when testing these sample materials. Therefore, the prevalence threshold of the biometrical study design was increased for these bioassays to 20%, while the probability for a type I error was kept at 5%. This required a minimum group size of 14 animals. On this basis, we i.c. injected three groups of (i) eight females and seven males, (ii) seven females and males each, and (iii) eight females and six males with 10% (w/v) of PBld, PStH and PMuH, respectively. Here, a negative test result (i.e., when in a group of at least 14 recipient animals none showed signs of exogenously stimulated αSyn aggregation) would be indicative, with an 80% confidence level, that the tested tissue contained at most 500 SD_50_ per gram.

### TgM83 bioassay

Three breeder pairs of hemizygous TgM83^+/−^ mice-expressing human αSyn with the A53T mutation maintained on a Bl6/C3 background (stock number 004479) were purchased from The Jackson Laboratory (Bar Harbor, USA). Homozygous TgM83^+/+^ mice, heterozygous TgM83^+/−^ mice and wild-type mice with Bl6/C3 background were generated by crossing hemizygous TgM83^+/−^ mice. Genotyping was performed by Taqman quantitative PCR run on an ABI 7500 (Thermo Fisher Scientific, Waltham, USA) corresponding to the genotyping protocol for these mice (stock number 004479; The Jackson Laboratory, Bar Harbor, USA) with the exception of using the fluorophore/quencher combinations YAK/BBQ for primer TmoIMR0105 and 6FAM/BBQ for primer TmoIMR0025.

Hemizygous TgM83^+/−^ mice have been reported to congenitally develop pathological αSyn deposits in the brain and severe motor neuron disease starting only from 22 months, i.e., 660 days, of age (see homepage from The Jackson Laboratory: https://www.jax.org/strain/004479). TgM83^+/−^ mice are highly susceptible to pathological effects of brain homogenates from patients with multi system atrophy (MSA), another human αSyn aggregation disease. I.c. injected brain tissue homogenates from MSA patients stimulated prominent deposits of pathologically aggregated αSyn within neuronal cell bodies and axons as well as severe motor disease after incubation periods of 106–144 dpi in TgM83^+/−^ mice [29, 39].

After anesthesia by inhalation exposure to isoflurane and subsequent intraperitoneal injection of a mixture of ketamine (100 mg/kg bodyweight) and xylazine (10 mg/kg bodyweight), 7- to 9-week-old hemizygous TgM83^+/−^ mice were challenged by i.c. injection of 20 μl 10% (w/v) homogenates in sterile PBS of the following tissues: (1) whole brain from a 288-day-old TgM83^+/+^ mouse with a pronounced motor disease phenotype [16]; (2) whole brain from an age matched wild-type mouse with Bl6/C3 background, (3) caudate nucleus from control donor 1 (NBH); (4) and (5) caudate nucleus from PD patient A and B (i.e., PBH A and PBH B), respectively; (6) and (7) skeletal muscle (biceps) and stomach wall from PD patient A (i.e., PMuH and PStH), respectively. A further group of TgM83^+/−^ mice was challenged by i.c. injection of 20 μl aliquots of whole blood from PD patient A (PBlH). All mice were implanted under anesthesia with a RFID-chip that assigned a unique identity number (ID) to each animal.

Mice were daily assessed for signs of neurological disease typical for the TgM83 model including tremor, ataxia and paralysis of one or more limbs [16, 39], as well as for other signs of health impairments such as weight loss, tension, nervousness, apathy or abnormalities in respiration, coat condition, digestion, or the intake of food and water. Animals were humanely euthanized when they reached a humane endpoint according to a clinical evaluation score sheet, or otherwise after incubation periods of 510–612 dpi.

After death mice were either transcardially perfused with periodate lysine paraformaldehyde for subsequent removal and paraffin-embedding of the brain, or the brain was removed without perfusion and immediately snap-frozen in liquid nitrogen and stored at − 80 °C. Where required, frozen brains were postfixed in 4% (w/v) formalin in PBS and subsequently embedded in paraffin. To preserve the morphologic tissue, structure-frozen tissues were slowly defrosted prior to postfixation.

### Immunohistochemistry

Immunostaining for αSyn detection was performed on paraffin-embedded coronal tissue sections (6 µm, cut from blocks equilibrated to room temperature [RT]) at three different sectional planes: (1) in the midbrain at the level of the substantia nigra, (2) in the midbrain at the level of raphe nuclei/periaqueductal gray, and (3) in the medulla oblongata at the level of vestibular nuclei. At these sectional planes, entire brain sections were scrutinized for staining signals of pathological αSyn phosphorylated at serine 129 (pSer129 αSyn) in perikarya and processes of cells. At least six sections from each mice and sectional plane were examined.

Antigen retrieval was done by boiling sections two times for 5 min. in 10 mM citric acid at pH 6.0 in a microwave oven. Endogenous peroxidases were inactivated by 3% (v/v) hydrogen peroxide in methanol for 20 min. Sections were subsequently treated with 1 µg/ml proteinase K (PK; Roche, Basel, Switzerland) in PBS (pH 7.0) for 15 min. at RT to reduce background staining. After blocking with blocking solution (0.2% [w/v] casein in PBS) two times for 10 min., sections were incubated with the primary antibody (monoclonal anti-pSer129 αSyn antibody ab51253; Abcam, Cambridge, UK) diluted 1:2500 in blocking solution containing 0.2% (w/v) Triton X-100 for 60 min. at 37 °C. After washing three times for 5 min. in PBS (pH 7.4), sections were incubated for 40 min. at RT in blocking solution containing biotinylated goat anti-rabbit IgG (E0432; Agilent Dako, Santa Clara, USA) at a dilution of 1:1000. Antibody labeling of pSer129 αSyn was prepared by applying the Vectastain ABC peroxidase kit (Vector Laboratories, Burlingame, USA) for 30 min. at RT according to the instructions of the manufacturer, and visualization of immunolabelling was done by exposure to 3,3′-diaminobenzidine for 3–5 min. Subsequently sections were counterstained with haematoxylin for 20 s (sec.), followed by a differentiation with 1% (w/v) HCl in 70% (v/v) EtOH and blue-staining in tap water. Slides were light-microscopically examined and photographed using an ICC50 HD (Leica, Nussloch, Germany) or Axioscope 40 (Zeiss, Feldbach, Switzerland) microscope.

### Haematoxylin and eosin staining

After rehydration, paraffin-embedded tissue sections (6 µm) were incubated for 10 min. in ready-to-use haematoxylin solution (Merck, Darmstadt, Germany) and differentiated in 1% (w/v) HCl in 70% (v/v) ethanol for 2 s. Staining was completed by incubation in tap water for 10 min., followed by counterstaining with 1% (w/v) eosin solution.

### Western blotting

To detect pathological insoluble high molecular weight (hMW) αSyn^PD^ aggregates in human tissue samples, we applied a Western blot assay the principle of which has been outlined by Kramer et al. [22] and Thomzig et al. [37]. This Western blot assay allows the specific detection of insoluble, highly aggregated αSyn species without prior sample treatment with proteinase K or other proteases. Typically, in this Western blot assay, the majority of highly aggregated PD-associated αSyn species remain insoluble after boiling in electrophoresis loading buffer, and such species are largely retained in the stacking gel.

**Fig. 1:**
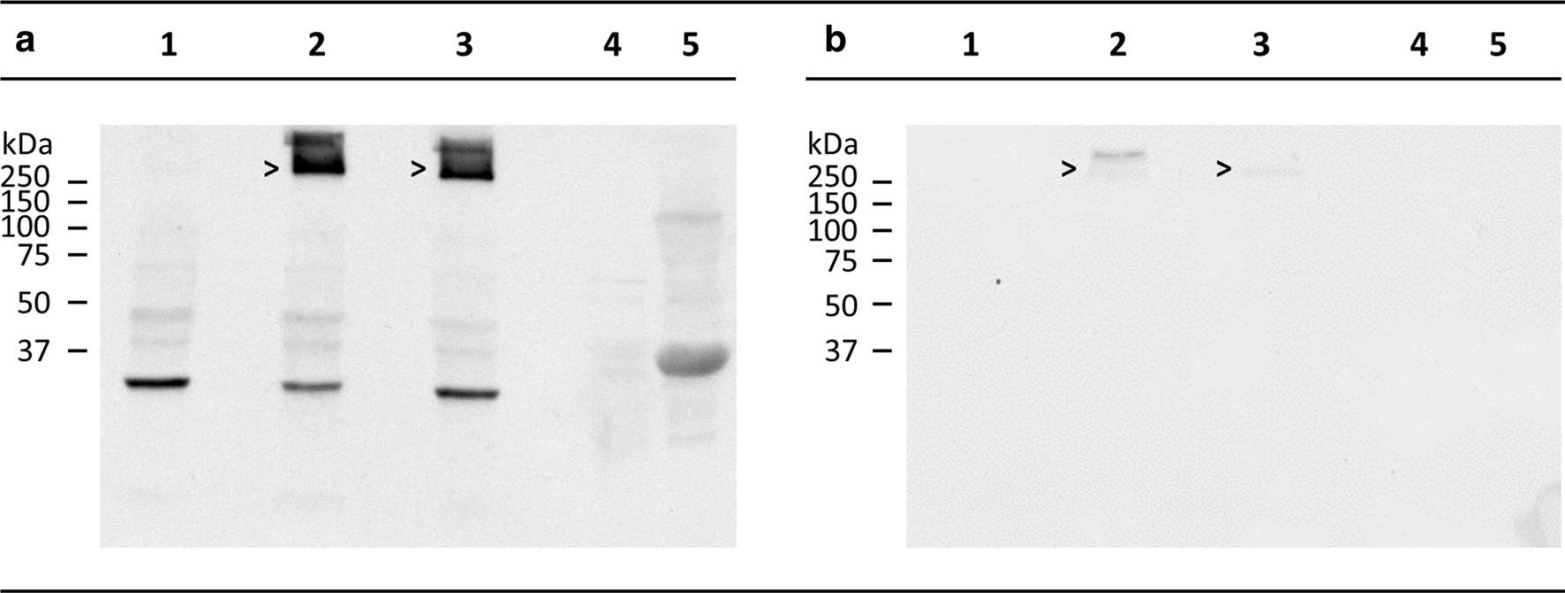
Western blot analysis of PD tissue homogenates for aggregated αSyn^PD^. **a**, **b** Insoluble high molecular weight (hMW) αSyn^PD^ aggregates could not be detected in NBH from a donor without PD (lane 1 each) but were clearly visible in PBH from PD patients A and B (lanes 2 and 3, respectively). Arrowheads indicate hMW αSyn^PD^ aggregates in a molecular weight range above 250 kDa, whereby these were specifically labeled for phosphorylated (pSer129) species in (**b**). Other than PBH, homogenates of stomach wall and skeletal muscle (biceps) from PD patient A did not show detectable species of hMW αSyn^PD^ aggregates (lanes 4 and 5, respectively). Distinct bands below 37 kDa in lanes 1–3 of Western blot **a** represented dimeric αSyn protein in NBH, PBH A and PBH B. Similar bands were not detectable in PStH or PMuH (lanes 4 and 5, respectively). Anti-αSyn antibody LB509 and anti-pSer129 αSyn antibody ab51253 were used for immunostaining in (**a**) and (**b**), respectively

In brief: 10% (w/v) tissue homogenates were prepared as described. 5 µl aliquots of 10% (w/v) tissue homogenates were mixed with 5 µl sample loading buffer (125 mM Tris pH 6.8, 20% glycerol [w/v], 10% mercaptoethanol [v/v], 4% SDS [w/v], 0.05% [w/v] bromophenol blue), incubated for 5 min. at 100 °C, and subjected to sodium dodecyl sulfate–polyacrylamide gel electrophoresis (SDS-PAGE) using 12.5% (w/v) SDS–polyacrylamide gels. After transfer of proteins from the stacking as well as the separating gel to polyvinylidene difluoride membranes (Immobilon; Merck Millipore, Darmstadt, Germany) using a semi-dry blotting apparatus (Biometra Fast blot, Analytik Jena, Jena, Germany) as previously described [36], membranes were blocked by incubation for 30 min. in Tris-buffered saline (TBS; 10 mM Tris–HCl, 133 mM NaCl, pH 7.4) containing 0.05% (w/v) Tween 20 (TBST) and 0.03% (w/v) casein (Casein–TBST). Blots were incubated overnight at 4 °C in primary antibody solution (monoclonal anti-αSyn antibody LB509 [Abcam, Cambridge, UK], diluted 1:5000 in Casein–TBST). Alternatively, blots were incubated for 1 h at RT in primary antibody solution containing monoclonal anti-pSer129 αSyn antibody ab51253 (Abcam, Cambridge, UK), diluted 1:5000 (for human sample material in Fig. 1) or 1:2500 (for positive TgM83^+/−^ mouse tissue in Fig. 3) in Casein–TBST. After washing five times for a total period of at least 20 min. with Casein–TBST, blots were incubated in secondary antibody solution containing alkaline-phosphatase-conjugated goat anti-mouse IgG (E0433; Agilent Dako, Santa Clara, USA) diluted 1:5000 in Casein–TBST for 90 min. at RT, or in secondary antibody solution containing alkaline-phosphatase-conjugated goat anti-rabbit IgG (D0487; Agilent Dako, Santa Clara, USA) diluted 1:5000 (for human sample material in Fig. 1) or 1:1000 (for positive TgM83^+/−^ mouse tissue in Fig. 3) in Casein–TBST for 40 min. at RT. After washing five times with Casein–TBST for a total period of at least 1.5 h, membranes were pre-incubated twice for 5 min. in buffer solution (100 mM Tris, 100 mM NaCl, pH 9.5) and developed with CDP-Star (Thermo Fisher Scientific, Waltham, USA) for 5 min. according to the instructions of the manufacturer. Signals were visualized using Hyper ECL film (Amersham Biosciences Corp., Waltham, USA).

### Real-time quaking-induced conversion (RT-QuIC) assay

The rationale of the RT-QuIC seeding activity assay is that PD- and other disease-associated αSyn aggregates are able to induce by seeded polymerization in vitro the conversion of monomeric recombinant αSyn (αSyn^rec^) into oligo- or polymeric aggregates rich in β-strand secondary structure. Vigorous periodic shaking (or “quaking”) is employed to stimulate this conversion reaction in various formats of well plates. Thioflavin T, a benzothiazole dye added to the reaction mixture, exhibits enhanced fluorescence and a characteristic red shift of its emission spectrum after binding to the amplified αSyn^rec^ aggregates. The resulting fluorescence signals indicate the occurrence and progress of aggregate amplification, i.e., the effect of αSyn seeding activity present in the test sample, and can be recorded in real time [9, 11, 26].

RT-QuIC assaying of stomach wall tissue for the detection of seeding activity triggering the aggregation of human αSyn^rec^ was performed in a pilot experiment using a modified protocol described by Pinder et al. [28]. In brief: 10% (w/v) PStH and NStH were either directly subjected to RT-QuIC analysis, or after 1:10 dilution in 10% (w/v) NBH to a final concentration of 1% (w/v). Examined samples consisted of 2 μl aliquots of 10% or 1% (w/v) PStH or NStH, added with either 2 μl 10% (w/v) PBH A or 10% (w/v) NBH. The homogenates were added into the wells of black polypropylene 96-well microplates containing 100 µl reaction mix (400 mM phosphate buffer [pH 8.0], 170 mM NaCl, 0.1 mg/mL recombinant human αSyn and 10 mM Thioflavin T [ThT]) and six glass beads (0.75–1.00 mm diameter). The plates were sealed using clear polyolefin sealing tape, placed inside an FLUOstar OPTIMA device and subjected to 1440 cycles of alternating incubation (1 min) and double orbital shaking (1 min) at 600 rpm over a time period of 48 h at 42 °C. Fluorescence intensity of ThT was measured from the top at 480 nm upon excitation at 450 nm. Maximum measurable fluorescence intensity of 65,000 was equated to 10,000 relative fluorescence units (rfu). Fluorescence readouts were analyzed and interpreted as previously described.

### Paraffin-embedded tissue (PET) blot

PET blot detection of aggregated and PK-resistant αSyn species in brain tissue sections from TgM83^+/−^ mice was performed on paraformaldehyde-fixed specimens as described previously [35], with modifications: after prewetting of blots with TBST adjusted to pH 7.8, sections were digested with 12.5 µg/ml PK in digestion buffer (10 mM Tris–HCl [pH 7.8], 100 mM NaCl, 0.1% Brij 35) for 1 h at 37 °C. Proteins attached to blot membranes were denatured with 3 M guanidine isothiocyanate in 10 mM Tris–HCl (pH 7.8) for 10 min. After preincubation in blocking solution (0.2% [w/v] casein in TBST, pH 7.8) for 30 min., blots were immunolabeled for 16 h at RT with anti-pSer129 αSyn antibody ab51253 diluted 1:2500 in blocking solution. Subsequently, blots were washed 3 × 10 min. in TBST (pH 7.8) and incubated for 60 min. at RT in alkaline phosphatase-coupled goat anti-rabbit antibody (E0432; Agilent Dako, Santa Clara, USA) at a dilution of 1:2000 in blocking solution. Visualization of antibody binding was achieved by staining with nitro blue tetrazolium/5-bromo-4-chloro-3-indolyl- phosphate (NBT/BCIP). PET blots were examined using a stereo microscope (Discovery V20; Zeiss, Feldbach, Switzerland).

## Results

To determine whether, and if so, which seeding effects i.c. injected PD tissue samples had in the TgM83^+/−^ model, we compared the cerebral deposition of phosphorylated pSer129 αSyn in and the clinical presentation of negative control, positive control and test mice. First, we determined the background of cerebral αSyn deposition and clinical features in sham-challenged TgM83^+/−^ mice that had been i.c. injected with NBH from a healthy wild-type mouse with Bl6/C3 background (*n* = 5) or from a human donor without neurodegenerative protein aggregation disease (*n* = 40). Any possible findings in test mice that should be attributed to the effect of injected exogenous αSyn^PD^ seeds had to differ significantly from the unspecific αSyn- or clinical background in our sham-challenged negative controls. As a second prerequisite for our bioassay study, we validated that our TgM83^+/−^ mice behaved after exposure to reference αSyn seeds in brain homogenate from clinically ill TgM83^+/+^-mice as previously reported [39]. Based on these characterizations of negative and positive controls, the actual bioassay testing of tissue samples from PD patients was then performed in TgM83^+/−^ reporter mice. In addition, all bioassayed PD tissue homogenates were characterized by Western blotting for the presence of aggregated αSyn^PD^. Furthermore, caudate nucleus as well as stomach wall tissue samples had been previously or were in this study, respectively, also scrutinized for αSyn^PD^ seeding activity after a suitable RT-QuIC assay had become available to us for this purpose.

### Characterization of tested tissues from PD patients

#### Brain

For Western blot analyses, two different anti-αSyn antibodies were used. Antibody LB509 did not discriminate between phosphorylated and non-phosphorylated forms of αSyn (Fig. 1a), whereas antibody ab51253 specifically labeled phosphorylated (pSer129) αSyn (Fig. 1b). No insoluble hMW αSyn^PD^ aggregates could be detected by Western blotting in caudate nucleus homogenate from control donor 1 (Fig. 1a, b—lane 1 each). In contrast, homogenates of caudate nucleus samples from PD patients A and B showed immunostaining signals in the stacking gel prominently featuring at the top of the Western blots in Fig. 1a, b (lanes 2 and 3 each; apparent MW ⪆ 250 kDa, marked by arrow; similar results as in Fig. 1b, lanes 2 and 3, were obtained for caudate nucleus tissue after immunolabelling with anti-pSer129 αSyn antibody pSyn #64 [WAKO, Neuss, Germany; not shown]). These signals originated from and indicated the presence of pathological hMW αSyn^PD^ aggregates [22, 37].

The caudate nucleus tissue from PD patients examined in these Western blots contained predominantly presynaptic αSyn^PD^ micro-aggregates instead of LBs, which are only phosphorylated at Ser 129 to a limited extent [33, 34]. This may provide an explanation for the relatively weak immunolabelling of hMW αSyn^PD^ aggregates by anti-pSer129 αSyn antibody ab51253 in Fig. 1b.

As determined in an RT-QuIC endpoint-titration experiment recently reported elsewhere, the caudate nucleus tissues from PD patients A and B contained an αSyn^PD^ seeding activity of 10^10.1^ and 10^10.0^ SD_50_ per gram of homogenized tissue, respectively [28]. This high level of αSyn^PD^ seeding activity substantiated, in retrospect, the suitability of the selected caudate nucleus tissue for testing in TgM83^+/−^ mice.

#### Skeletal muscle, stomach wall and blood

Frozen sample material from skeletal muscle (biceps) and the stomach wall of PD patient A was homogenized and also examined by Western blotting for hMW αSyn^PD^ aggregates. Other than for brain homogenate the Western blot analysis of these peripheral tissues provided negative results (Fig. 1a, b—lanes 4 and 5 each). A similar examination of whole blood from patient A by Western blotting was not successful because that sample material coagulated after boiling in electrophoresis sample buffer and could, therefore, not be subjected to SDS-PAGE.

The stomach wall homogenate was additionally analyzed retrospectively, in a pilot experiment, by RT-QuIC assay for in vitro detection of αSyn^PD^ seeding activity after our TgM83^+/−^ bioassay had provided positive results for PStH (see below) despite the negative Western blot findings obtained with this tissue. Since the donor of negative control NStH had Covid-19, his tissue sample was heated to 65 °C for 45 min to inactivate coronavirus SARS-CoV-2 for biosafety reasons. To match these tissue-processing conditions required for our negative control sample, a subset of PStH specimens was also subjected to the same heat treatment prior to RT-QuIC analysis.

**Fig. 2:**
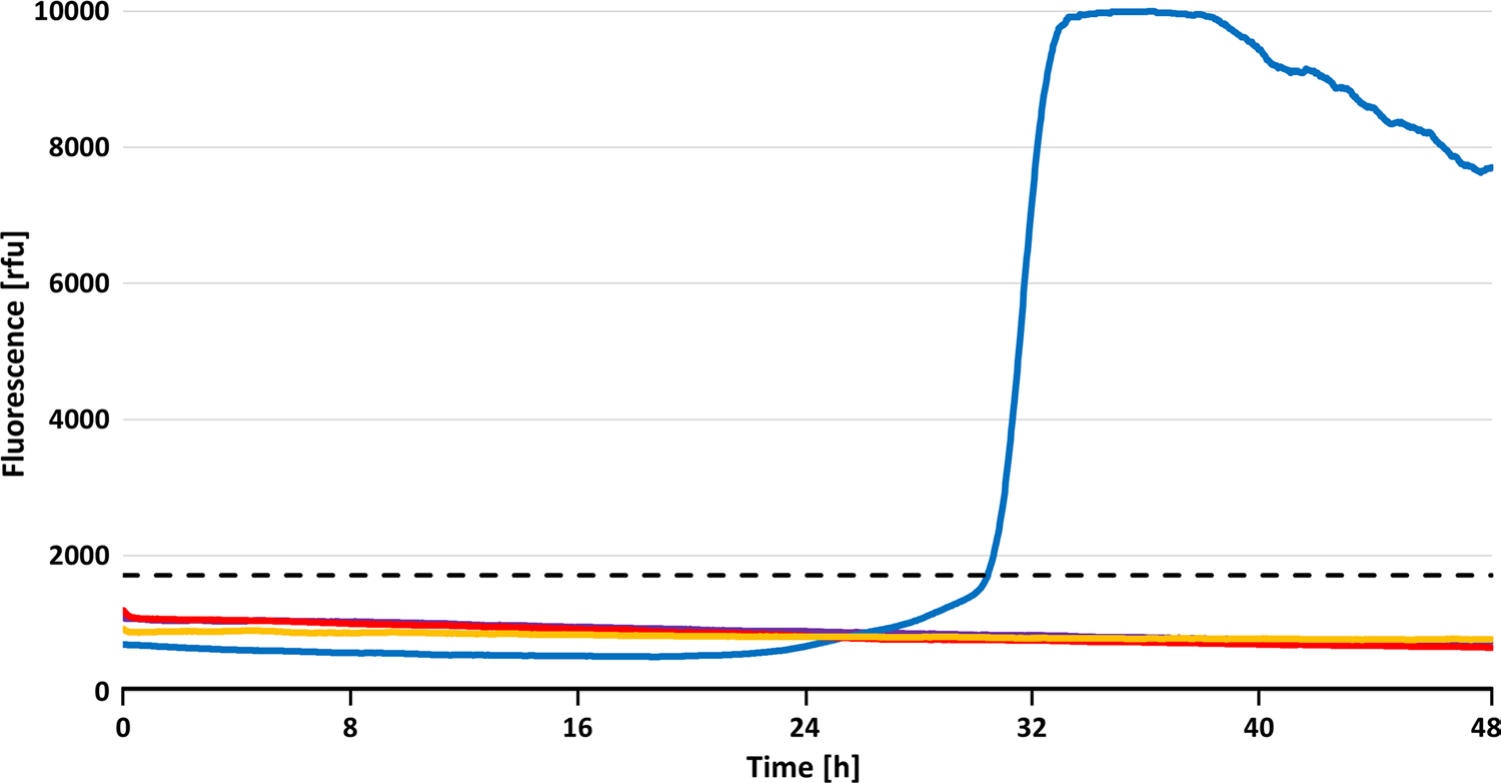
Pilot testing of stomach wall tissue from a PD patient for αSyn^PD^ seeding activity using real-time quaking-induced conversion (RT-QuIC) assay. RT-QuIC analysis of 10% (w/v) PStH and NStH did not allow detection of endogenous and interfered with detection of exogenously added PBH seeding activity. Dilution of stomach wall homogenates to 1% (w/v) abolished the interfering effect, and 1% (w/v) unheated PStH spiked with PBH showed readily detectable seeding activity. Under these experimental conditions, endogenous seeding activity was found in unheated or heated PStH, but not detected in heated NStH. Exemplary results from *n* = 10 test runs each are shown. Color code of samples: violet, 10% (w/v) PStH + 10% (w/v) NBH; red, 10% (w/v) NStH + 10% (w/v) NBH; blue, 1% (w/v) PStH + 10% (w/v) NBH; yellow, 1% (w/v) NStH + 10% (w/v) NBH. The examined stomach wall tissues represented in this figure had been heated to 65 °C for 45 min prior to homogenization to inactivate SARS-CoV-2 virus potentially present in the stomach wall tissue from control donor 2 and to match this processing conditions for PStH. Black dashed line marks cutoff value set for separation of seeding-negative from seeding-positive samples. *rfu* Relative fluorescence units

RT-QuIC analysis of unheated 10% (w/v) PStH that had been spiked with PBH revealed an inhibition of the detection of the exogenously added seeding activity in this sample material (Table 1). This indicated that an unknown component of 10% (w/v) stomach wall homogenate interfered with our RT-QuIC assay. Since this may have artefactually caused the negative test results observed for heated 10% (w/v) PStH and NStH (Table 1; Fig. 2—violet and red lines, respectively), we sought to counteract the interfering effect by lowering the concentration of the stomach wall homogenates to 1% (w/v). Under these experimental conditions, exogenously added PBH seeding activity could be detected in unheated PStH by our RT-QuIC assay (Table 1). At the same time, endogenous seeding activity was found in unheated as well as heated 1% (w/v) PStH, while ten negative control samples of heated 1% (w/v) NStH other than positive control samples tested on the same 96-well microplate consistently failed to show seeding activity (Table 1; Fig. 2—blue and yellow lines for heated 1% [w/v] PStH and NStH, respectively).

**Table 1:** Readouts of RT-QuIC assay with stomach wall tissue from a PD patient and a non-PD control donor

Conc. [%]	PStH		PStH (65 °C)	NStH (65 °C)
	+ NBH	+ PBH	+ NBH	+ NBH
10	0/10	0/10	0/10	0/5
1	10/10	10/10	10/10	0/10

Results of pilot testing expressed as number of RT-QuIC samples with a positive assay readout/number of RT-QuIC samples tested. As described previously [28], tissue samples were considered as positive for αSyn^PD^ seeding activity when four or more out of ten test runs delivered a positive RT-QuIC readout. Conc.: concentration (w/v) of tested PStH or NStH. NBH: 10% (w/v) non-PD brain homogenate (from control donor 1; 2 μl was added to the indicated reaction mixtures). N.d.: not determined. NStH (65 °C): non-PD stomach wall homogenate (from control donor 2; for biosafety reasons heated to 65 °C for 45 min. prior to RT-QuIC analysis to inactivate coronavirus SARS-CoV-2). PBH: 10% (w/v) PD brain homogenate (from PD patient A; 2 μl was added to the indicated reaction mixtures). PStH: PD stomach wall homogenate (from PD patient A). PStH (65 °C): PStH heated to 65 °C for 45 min. prior to RT-QuIC analysis (to match processing conditions of NStH [65 °C]). An unknown component of 10% (w/v) stomach wall homogenate interfered with our RT-QuIC assay. To counteract this interfering effect, the concentration of PStH and NStH was lowered to 1% (w/v)

Since the heated PStH and NStH samples had been treated equally prior to RT-QuIC analysis, the positive RT-QuIC readout for heated PStH is unlikely to have resulted from an unspecific non-αSyn^PD^ factor generally present in stomach wall tissue. Despite exposure to 65 °C prior to homogenization, seeding activity was readily detected in 1% (w/v) PStH, which is consistent with the high resistance of αSyn^PD^ seeds to heat inactivation [28]. Thus, our RT-QuIC results indicated in vitro the presence of αSyn^PD^ seeds in PStH from PD patient A.

### Characterization of the TgM83^+/−^ mouse model

#### Sham-challenged TgM83^+/−^ mice

Of 40 TgM83^+/−^ mice injected with human NBH, 5 did not reach the minimum incubation period for our bioassay of 400 dpi so that the readout of their IHC examination was unusable (Supplementary Table 1, online resource). This reduced the number of NBH sham-challenged mice available for IHC analysis to *n* = 35. In contrast, all of the animals sham-challenged with murine NBH (*n* = 5) were eligible to IHC analysis.

**Fig. 3:**
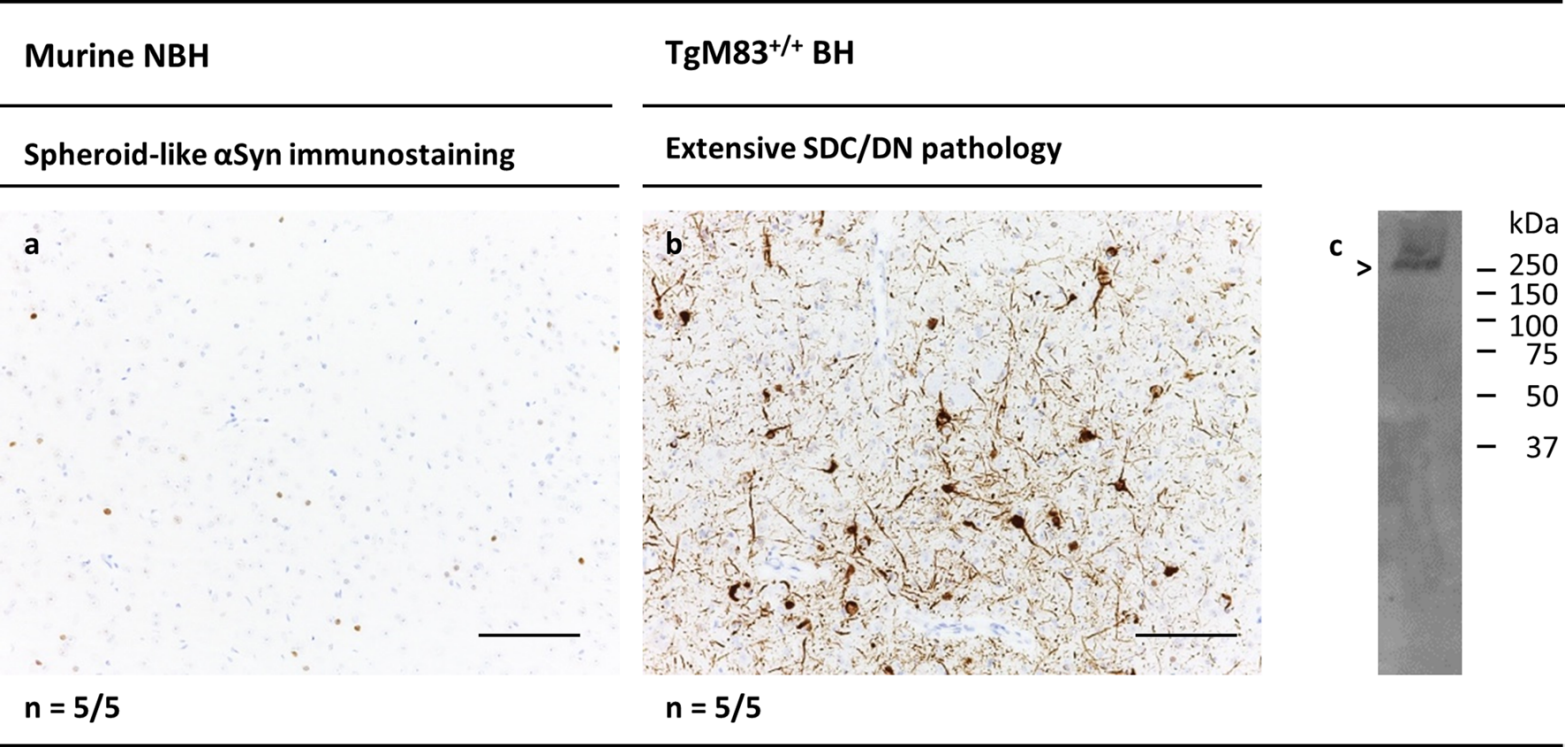
Cerebral αSyn deposition in TgM83^+/−^ mice challenged with brain homogenates from a normal control or diseased TgM83^+/+^ mouse. **a** Spheroid-like cerebral αSyn deposits exemplarily shown for the periaqueductal gray brain region of a TgM83^+/−^ mouse sham-challenged with NBH from a wild-type mouse with C57Bl6/C3 background (202 dpi). **b** Pronounced pathology of αSyn deposition in the somatodendritic compartment (SDC) and dystrophic neurites (DNs), referred to as extensive (or phenotype “E”) SDC/DN pathology, exemplarily shown for the periaqueductal gray region of a clinically ill TgM83^+/−^ mouse challenged with brain homogenate from a 288-day-old TgM83^+/+^ donor mouse with model-typical motor disease (198 dpi). Bars: 100 µm. *n* number of animals showing the indicated profile of cerebral αSyn deposition/number of animals examined. **c** Western blot detection of insoluble hMW αSyn aggregates in brain homogenate from a TgM83^+/−^ mouse challenged with the same inoculum as in (**b**) (205 dpi). Arrowhead indicates phosphorylated (pSer129) hMW αSyn aggregates in a molecular weight range above 250 kDa. Anti-pSer129 αSyn antibody ab51253 was used for IHC and Western blot staining**.** *BH* Brain homogenate; *n* number of animals showing the indicated profile of cerebral αSyn deposition/number of animals examined; *NBH* Non-PD brain homogenate; *SDC/DN* somatodendritic compartment/dystrophic neurite

**Fig. 4:**
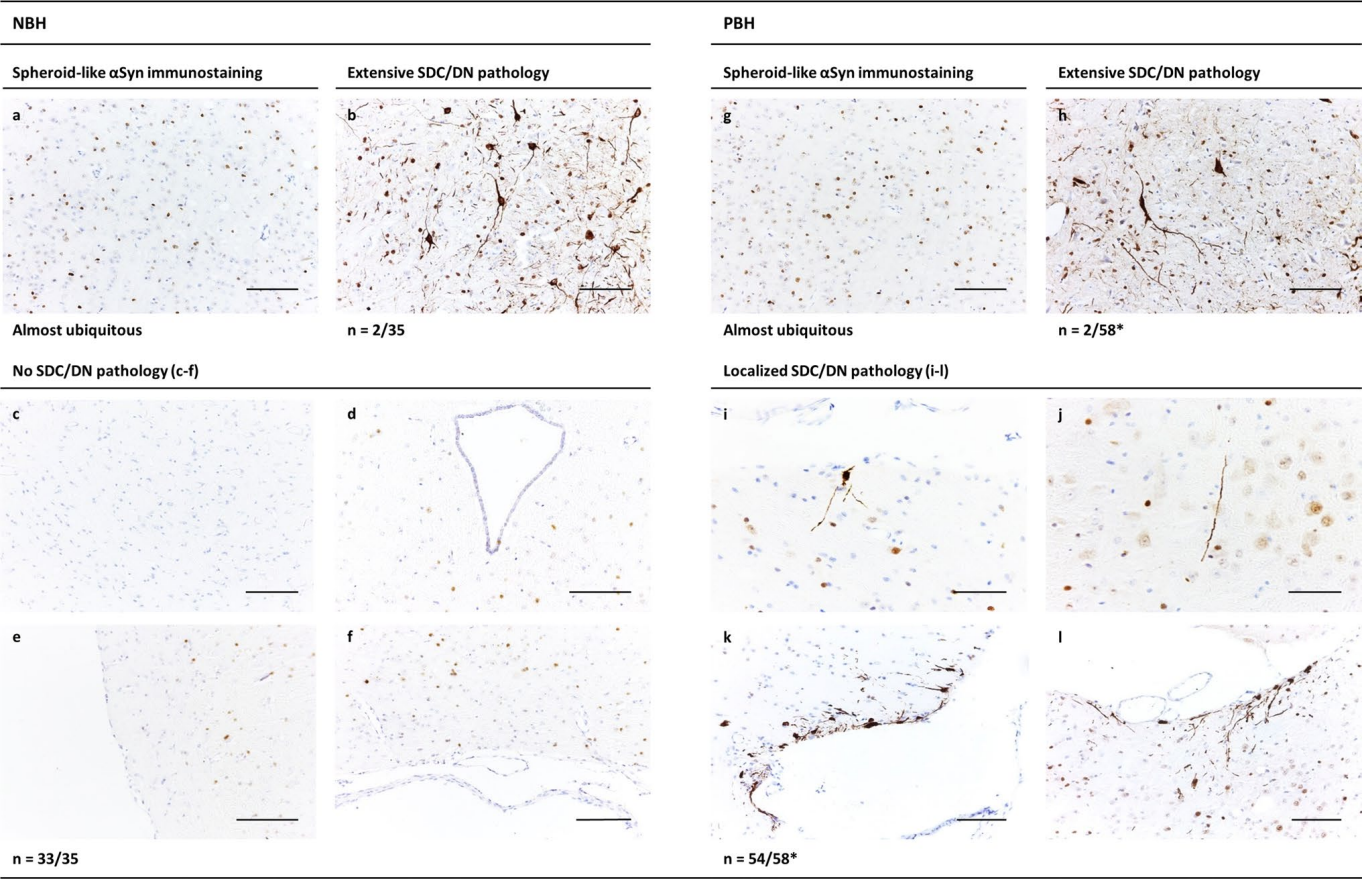
Cerebral αSyn deposition in TgM83^+/−^ mice challenged with human brain homogenates from a non-PD control donor or PD patients. **a**, **g** Spheroid-like cerebral αSyn deposits exemplarily shown for the periaqueductal gray brain region, in TgM83^+/−^ mice sham-challenged with human NBH (ID 7074, 571 dpi) or challenged with PBH A (ID 4851, 580 dpi), respectively. This phenotype of immunostaining occurred almost ubiquitously (as far as not masked by extensive SDC/DN pathology) at the examined different sectional planes of TgM83^+/−^ mice, and seemed to became more pronounced with increasing age. **b**, **h** Extensive (or “E”) SDC/DN pathology exemplarily shown for the periaqueductal gray region after sham-challenge with human NBH (ID 8011, 516 dpi) or challenge with PBH B (ID 3154, 469 dpi), respectively. Extensive SDC/DN pathology was observed in 2 out of 35 examined mice sham-challenged with human NBH as well as in 2 out of 58 examined mice challenged with PBH. **c**–**f** Absence of SDC/DN pathology in any of the brain slices examined from control mice that had been sham-challenged with NBH and not developed the extensive phenotype of SDC/DN pathology. Exemplary findings representatively illustrating the consistent lack of SDC/DN pathology observed in 33 out of 35 examined sham-challenged mice: **c** Midbrain—substantia nigra pars compacta (ID 0497, 570 dpi); **d** midbrain—periaqueductal gray (ID 5443, 571 dpi); **e** midbrain—cerebral peduncle/substantia nigra reticulata (ID 2776, 573 dpi); **f** medulla oblongata—pyramidal/reticular nuclei (ID 7936, 567 dpi). **i**–**l** Localized (or “L”) SDC/DN pathology in the brain of TgM83^+/−^ mice challenged with PBH A or B confined to isolated individual nerve cells or DNs in the examined brain slices (sub-phenotype L+ of SDC/DN pathology), or detectable in a few separated areas, which contained several stained neurons or DNs (sub-phenotype L++ of SDC/DN pathology). Shown are exemplary findings for both sub-phenotypes from four different mice free of neurological symptoms: **i** L+ in midbrain—mammillary nucleus/cerebral peduncle (ID 4384, 570 dpi); **j** L+ in midbrain—reticular nucleus (ID 6689, 572 dpi); **k** L++ in midbrain—mammillary nucleus/cerebral peduncle (ID 6229, 572 dpi); **l** L++  in medulla oblongata—pyramidal/reticular nuclei (ID 6445, 572 dpi). Anti-pSer129 αSyn antibody ab51253 was used for immunohistochemical staining. Bars: 50 µm in (**i**, **j**); 100 µm in (**a**–**h**,** k**, **l**). *n* number of animals showing the indicated profile of cerebral αSyn deposition/number of animals examined; *NBH* non-PD brain homogenate; *PBH* PD brain homogenate; *SDC/DN* somatodendritic compartment/dystrophic neurite. *Two out of 58 mice with incubation periods of > 400 dpi showed no SDC/DN pathology, neither localized nor extensive

Examined brain sections from the TgM83^+/−^ mice injected with murine or human NBH almost ubiquitously showed a background staining of spots of spheroid-like αSyn immunolabelling in varying numbers and intensities (Figs. 3a and 4a). Our immunohistochemical analyses suggested that this type of αSyn immunostaining became more pronounced with increasing age (not shown).

TgM83^+/−^ mice were reported to develop severe motor impairments (e.g., partial paralysis of one or more limbs) along with pronounced widespread cerebral inclusions of pathologically aggregated αSyn in the somatodendritic compartment (SDC) and dystrophic neurites (DNs) either congenitally at ≥ 660 days of age (see homepage from The Jackson Laboratory: https://www.jax.org/strain/004479) [16], or due to injected seeding-active αSyn species earlier in life [29, 39]. That model-typical profile of pathological αSyn deposition in the brain (for examples, see Fig. 4 in [39]) is referred to in this report as extensive (or phenotype “E'”) somatodendritic/dystrophic neurite (SDC/DN) pathology. As expected, we did neither observe this pathological phenotype nor motor impairments in the five relatively young sham-challenged TgM83^+/−^ mice exposed to murine NBH which were examined at incubation times of 180–219 dpi and an age of 231–272 days. However, when we examined the 35 TgM83^+/−^ mice that had been injected with human NBH and killed after 456–612 dpi within an age-range of between 511 and 654 days, we found that 2 (6%) of these negative control animals showed extensive SDC/DN pathology (Fig. 4b), while the other 33 control mice of this group did not show any detectable pathological αSyn deposition in the SDC (i.e., simultaneously in the soma and dendrites of neurons) or DNs (Fig. 4c–f). Notably, the age of the two negative control animals with extensive SDC/DN pathology was only 566 and 581 days (IDs 8011 and 9066; Supplementary Table 1, online resource).

Of the 35 sham-challenged TgM83^+/−^ mice of this negative control group, 3 (9%) developed severe neurological disease with motor impairments at an age of 513, 526 and 566 days (IDs 6793, 5464 and 8011, respectively; Supplementary Table 1, online resource). While only one of these animals (ID 8011; Supplementary Table 1, online resource) also exhibited extensive SDC/DN pathology, another mouse of this group (ID 6793; Supplementary Table 1, online resource) had presumably suffered an ischemic stroke, as indicated by a lens-shaped blood clot in the brain stem.

#### TgM83^+/−^ mice injected with αSyn seeding activity from TgM83^+/+^ donor animals

I.c. injection of brain homogenate from a clinically ill TgM83^+/+^ mouse (euthanized at an age of 288 days) in five 7- to 9-week-old TgM83^+/−^ mice resulted in an accelerated onset of pronounced neurological symptoms typical for this experimental paradigm [39] in the recipient animals at 178–219 dpi (median: 201 dpi; mean ± standard deviation [SD]: 201 ± 16 dpi) and 226–267 days of age (median 244 days; mean ± SD 245 ± 16 days). Immunostaining of brain sections for detection of αSyn deposition revealed dense, intense and widespread labeling of pathologically aggregated αSyn in neuronal perikarya and processes as well as DNs (Fig. 3b) in all of these mice. Thus, these mice consistently showed the extensive SDC/DN pathology previously observed by others in homozygous or hemizygous TgM83 mice challenged with seeding-active human αSyn species from TgM83^+/+^ mice or MSA patients, respectively [39].

Extensive SDC/DN pathology as well as pronounced neurological symptoms occurred with statistical significance more frequently in the mice challenged with TgM83^+/+^ brain homogenate (five neuropathologically and clinically positive animals out of five examined animals) than in age- and incubation time-matched mice injected with murine NBH (zero neuropathologically or clinically positive animals out of five examined animals; *p* = 0.0079 each according to Fisher´s exact test). Thus, our validation experiment recapitulated consistently the previously reported observations, and the conclusion that the stimulation of extensive SDC/DN pathology as well as of disease symptoms could be attributed to the αSyn seeding activity present in the tested TgM83^+/+^ brain homogenate.

### Analysis of cerebral αSyn deposition and clinical symptoms in TgM83^+/−^ test mice

#### TgM83^+/−^ mice challenged with brain tissue from PD patients

Two out of 60 TgM83^+/−^ mice challenged with PBH did not reach the minimum incubation period for our bioassay of 400 dpi (Supplementary Table 1, online resource). Since the readout of their IHC examination was, therefore, unusable, this reduced the number of PBH-challenged mice available for IHC analysis to *n* = 58.

TgM83^+/−^ mice that had been i.c. inoculated with PBH almost ubiquitously showed a background of spheroid-like αSyn immunostaining (Fig. 4g) which was undistinguishable from that observed in sham-challenged TgM83^+/−^ of similar age (Fig. 4a). Accordingly, an effect of αSyn^PD^ seeds on the development of this type of αSyn deposition in TgM83^+/−^ mice could not be detected.

In addition, the occurrence of extensive SDC/DN pathology was practically non-different from that in the sham-challenged control group. After injection of PBH from patients A and B we observed this pathology in none out of 30 and in 2 out of 28 challenged mice examined at ≥ 400 dpi, respectively (Fig. 4h; Supplementary Table 1, online resource). The age of the two test mice showing this neuropathological phenotype after inoculation with PBH B was 522 and 602 days. Thus, extensive SDC/DN pathology did not occur, with statistical significance, more frequently or earlier in TgM83^+/−^ mice injected with PBH than in control animals sham-challenged with NBH. Accordingly, from the two observed cases of extensive SDC/DN pathology in the test group, no evidence for an αSyn^PD^ seeding effect of the injected PD brain homogenates could be derived. The extensive SDC/DN pathology had to be considered as unspecific in our experimental setup with respect to injected αSyn^PD^ seeds and might also have interfered with the detection of other, more subtle forms of αSyn aggregation that possibly also occurred in our bioassay animals. To avoid confounding effects, PBH- as well as NBH-challenged mice showing this profile of extensive cerebral αSyn deposition were, therefore, excluded from the statistical analysis for the detection of αSyn^PD^ seeding (Supplementary Table 1, online resource). This resulted in group sizes of PBH- and NBH-challenged TgM83^+/−^ mice available for this purpose of *n* = 56 and 33, respectively (Table 2).

**Table 2:** Statistical analysis of immunohistochemical findings on localized SDC/DN pathology in the brain of TgM83^+/−^ test mice

Sex of mice	Inoculum	*N*	Incubation period		Age		Localized SDC/DN pathology^a^	*P*
			Median	Mean	Median	Mean		
Male	NBH	13	572	552	621	601	0/13	n. a
	PBH A	15	580	582	627	630	15/15	< 0.00001*
	PBH B	11	571	546	623	598	10/11	< 0.00001*
	PStH A	7	546	542	595	599	3/4	0.0307*
Female	NBH	20	570	563	621	614	0/20	n. a
	PBH A	15	572	545	620	592	15/15	< 0.00001*
	PBH B	15	570	560	620	612	14/15	< 0.00001*
	PStH A	7	541	546	595	599	4/3	0.002*
Male and female	NBH	33	570	559	621	608	0/33	n. a
	PBH A	30	572	563	622	611	30/30	< 0.00001*
	PBH B	26	570	550	620	602	24/26	< 0.00001*
	PStH A	14	546	542	595	599	7/7	0.0001*

Localized SDC/DN pathology is characterized by the cerebral deposition of pathologically phosphorylated and aggregated αSyn in individual or focally clustered somatodendritic compartments or DNs. DN(s): dystrophic neurite(s). *n* number of animals. ^a^ *n*/*n*_0_ number of animals with localized SDC/DN pathology/number of examined animals. n. a.: not applicable. *p*: significance according to Fisher’s exact test (vs. NBH)

*NBH* non-PD brain homogenate. *PBH A or B* PD brain homogenate from PD patient A or B, respectively. *PStH A* homogenate of stomach wall tissue from PD patient A. *SDC* somatodendritic compartment

*: *p* < 0.05

Indeed, the immunohistochemical examination of brain sections from the 56 TgM83^+/−^ mice challenged with PBH A or B that had been killed between 404 and 612 dpi at an age of 455–658 days and did not show extensive SDC/DN pathology unexpectedly revealed a more subtle yet consistent deposition of pathologically aggregated αSyn in the SDC and DNs. This phenotype of αSyn deposition showed an isolated or focal occurrence often at the interface of neuronal brain tissue with the pia mater or ventricles, and is referred to in this report as localized (or phenotype “L”) SDC/DN pathology. Immunostaining of this pathological phenotype was either confined to isolated individual nerve cells or DNs in the brain slices (sub-phenotype L+ of SDC/DN pathology; Fig. 4 i, j; Supplementary Table 1, online resource), or detectable in a few separated areas, which contained several stained neurons or DNs (sub-phenotype L++ of SDC/DN pathology; Fig. 4 k, l; Supplementary Table 1, online resource). Remarkably, localized SDC/DN pathology was not detected in any of the brain slices examined from the 33 control mice that had been sham-challenged with NBH and euthanised for neuropathological analysis between 456 and 612 dpi at an age of 511–654 days without having developed extensive SDC/DN pathology (Fig. 4c–f; Supplementary Table 1, online resource).

Brain sections from the medulla oblongata generally showed easily detectable and relatively prominent localized SDC/DN pathology in PBH-challenged mice, so that the contrast with the negative findings in NBH sham-challenged control mice is particularly striking. Supplementary Fig. 1, online resource, juxtaposes in ten paired examples typical findings on SDC/DN pathology in the medulla oblongata of TgM83^+/−^ mice that had been exposed to NBH or PBH for more detailed documentation of the appearance of negative and positive IHC stainings.

**Fig. 5:**
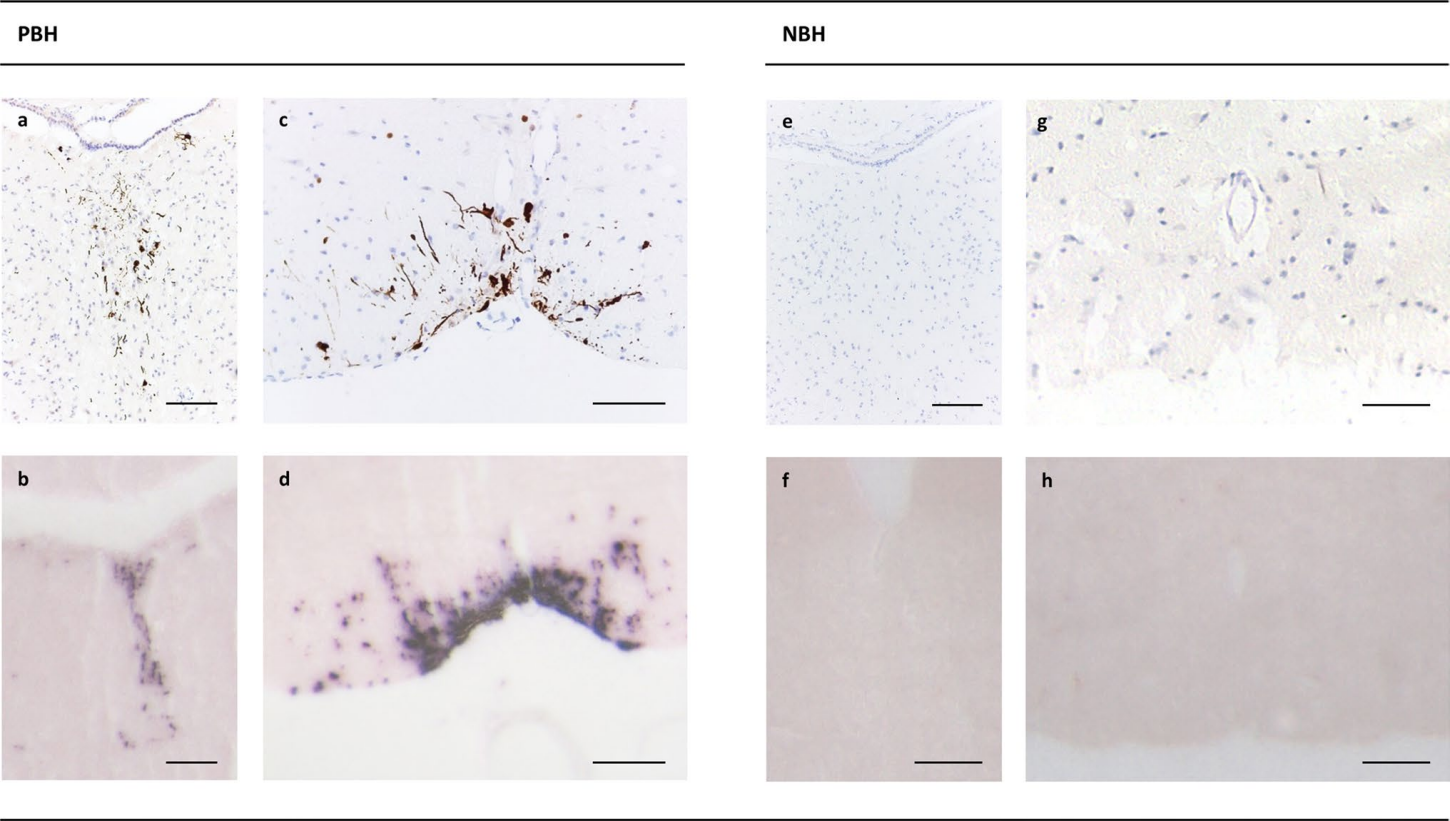
Comparative immunohistochemical and PET blot analysis of brain sections from TgM83^+/−^ mice challenged with homogenates of PD or non-PD control brain tissue. **a**–**d** Brain areas found by immunohistochemistry to exhibit localized SDC/DN pathology (upper panel) in mice challenged with PBH were also immunostained in PET blots (lower panel) after exposure to Proteinase K (PK), to confirm that the immunohistochemical staining of αSyn represented pathological PK-resistant αSyn aggregates. Shown are the following exemplary findings from two animals: **a**, **b** immunohistochemical and PET blot staining, respectively, in pontine central gray at 4th ventricle of the medulla oblongata (ID 2037, 553 dpi); **c**, **d** immunohistochemical and PET blot staining, respectively, in pyramidal/reticular nuclei of the medulla oblongata (ID 0763, 571 dpi). **e**–**h** Brain areas found by immunohistochemistry to display no SDC/DN pathology (upper panel) in mice sham-challenged with NBH were also examined by sensitive PET blotting (lower panel) to confirm the absence of pathological PK-resistant αSyn deposits. Exemplary findings from one animal (ID 0497, 570 dpi) are shown: **e**, **f** immunohistochemical and PET blot staining, respectively, in pontine central gray at 4th ventricle of the medulla oblongata; **g**, **h** immunohistochemical and PET blot staining, respectively, in pyramidal/reticular nuclei of the medulla oblongata. Anti-pSer129 αSyn antibody ab51253 was used for immunohistochemical and PET blot staining. Bars: 100 µm. *NBH* non-PD brain homogenate; *PBH* PD brain homogenate

To validate that the immunohistochemically detected localized SDC/DN pathology represented deposits of pathologically aggregated αSyn that were resistant to degradation by proteases, a hallmark of many pathologically aggregated protein species of different protein aggregation diseases, we also subjected brain slices from PBH-challenged TgM83^+/−^ mice to PET blot analysis. Brain areas found by immunohistochemistry to exhibit localized SDC/DN pathology (Fig. 5a, c) were also immunostained in PET blots (Fig. 5b, d, respectively) after exposure to 12.5 µg/ml PK. This confirmed that the immunohistochemical staining of localized SDC/DN pathology represented PK-resistant αSyn aggregates. Figure 5b, d demonstrate the sensitivity of the PET blot method for the detection of pathological αSyn deposits. On this basis, we used PET blotting also for confirming the absence of pathological αSyn deposition indicated by IHC in negative control mice, as exemplified in Fig. 5e–h for pontine central gray at 4th ventricle and pyramidal/reticular nuclei of the medulla oblongata from an NBH-challenged animal.

The statistical analysis of immunohistochemical findings summarized in Table 2 formally confirmed that the localized phenotype of SDC/DN pathology (i.e., L+ or L++) occurred in TgM83^+/−^ test mice injected with PBH A or PBH B with statistical significance more frequently than in sham-challenged control animals. This applied both when the comparison for male and female animals was carried out separately or in combination (*p* < 0.00001 for all performed tests). These findings strongly suggest that the injected PBH from both patients consistently stimulated a subtle cerebral aggregation and deposition of human αSyn in male and female TgM83^+/−^ mice, thereby leading to the development of the localized phenotype of SDC/DN pathology.

Despite the obvious stimulation of aggregation and deposition of endogenously expressed human αSyn by i.c. injected PBH in the brain of TgM83^+/−^ mice, we failed to detect neurological or other disease symptoms that could be associated with the observed seeding effects of αSyn^PD^. Similarly to the negative control group only three cases of neurological disease with movement impairment were observed in test mice at an age of 511, 566 and 602 days (IDs 3166, 4848 and 9907, respectively; Supplementary Table 1, online resource). Again, only one of these animals (ID 9907; Supplementary Table 1, online resource) showed extensive SDC/DN pathology. Thus, the clinical monitoring of our study animals over long incubation times up to 612 dpi did not provide evidence for a higher incidence or faster onset of neurological impairments in TgM83^+/−^ test mice than in negative control animals.

#### TgM83^+/−^ mice challenged with blood, muscle or stomach wall tissue of a PD patient

**Fig. 6:**
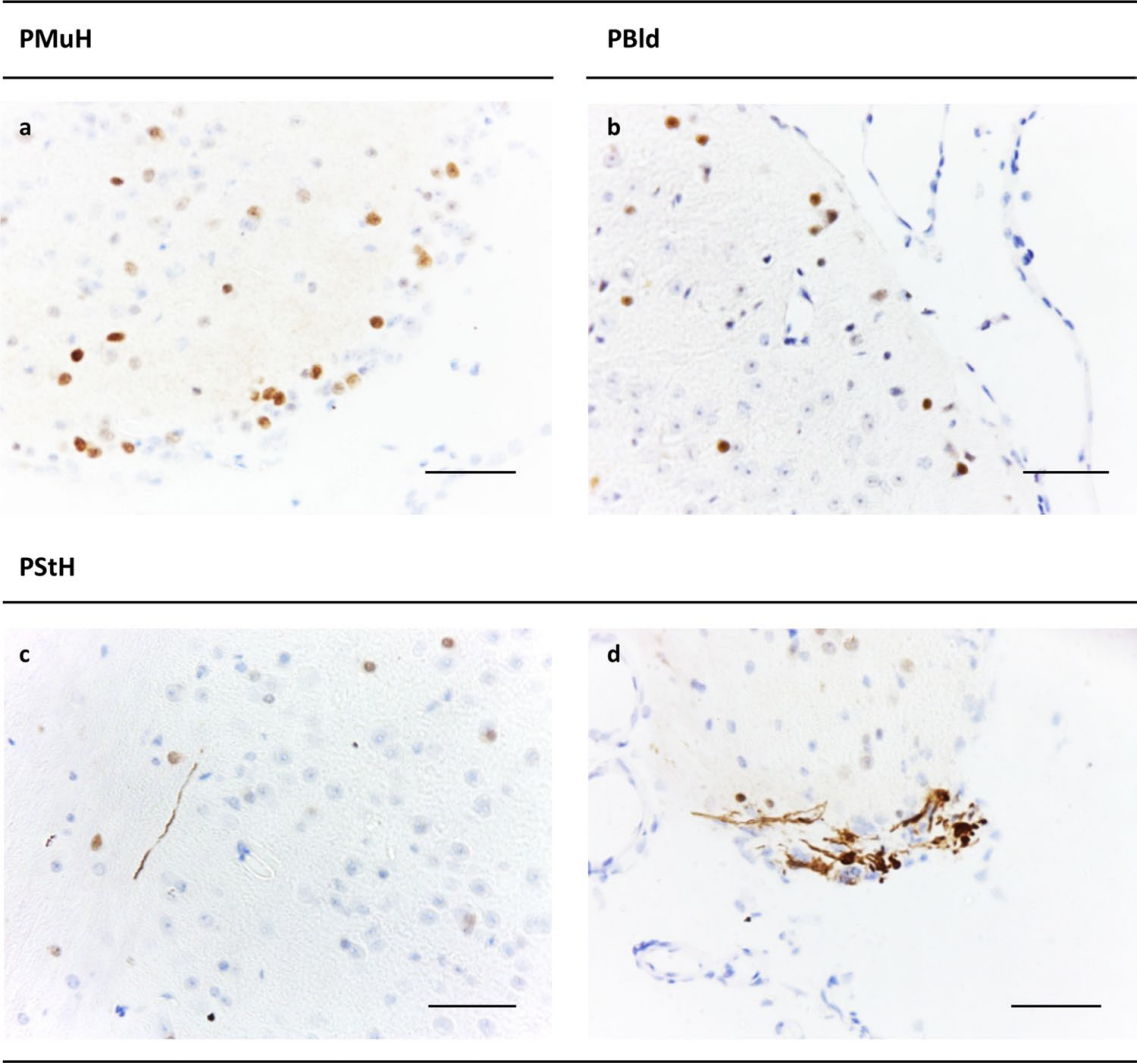
Immunohistochemical analysis of cerebral αSyn pathology in TgM83^+/−^ mice challenged with stomach wall, skeletal muscle or blood of a PD patient. **a**, **b** Brain sections from TgM83^+/−^ mice challenged with whole blood or homogenized skeletal muscle (biceps) from PD patient A showed only unspecific spheroid-like αSyn immunostaining also found in sham-challenged controls (Fig. 4a). Shown are exemplary findings from the midbrain (cerebellar peduncle): **a** mouse challenged with PMuH (ID 61550, 551 dpi); **b** mouse challenged with PBld (ID 59880, 518 dpi). **c**, **d** TgM83^+/−^ mice challenged with PStH also showed such spheroid-like αSyn immunostaining, but 50% of these mice additionally exhibited localized SDC/DN pathology as displayed in exemplary findings from the midbrain of two mice: **c** L+ in cerebellar peduncle (ID 75523, 566 dpi); **d** L++ in reticular nucleus (ID 58542, 524 dpi). Anti-pSer129 αSyn antibody ab51253 was used for immunohistochemical staining. Bars: 50 µm. PBld, PMuH, PStH: blood, muscle homogenate and stomach wall homogenate from PD patient A, respectively

TgM83^+/−^ mice which had been injected with homogenates of skeletal muscle (*n* = 6 females and six males; two out of eight female mice challenged with PMuH died immediately after i.c. injection without waking up and were, therefore, excluded from the analysis) or whole blood (*n* = 8 females and seven males) from PD patient A did not show pathological αSyn aggregation and deposition in their brains (Fig. 6a, b) with the exception of one blood-challenged animal. This mouse developed the extensive phenotype of SDC/DN pathology at 517 dpi aged 571 days (ID 55565; Supplementary Table 1, online resource) and was excluded from further statistical analysis.

The group of TgM83^+/−^ mice i.c. injected with tissue homogenate from the stomach wall of PD patient A comprised seven female and seven male animals. Of these animals, four females developed localized SDC/DN pathology of sub-phenotype L+ (Fig. 6c). In addition, we observed the sub-phenotype L++ of localized SDC/DN pathology in one female and two male animals of this group (Fig. 6d). Thus, 7 out of 14 mice challenged with the stomach tissue homogenate showed localized SDC/DN pathology. Accordingly, this pathology occurred in TgM83^+/−^ test mice injected with this sample material significantly more frequently than in the control animals that had been sham-challenged with NBH (Table 2). That was the case independently of whether the comparison for male and female animals was carried out separately (*p* = 0.0307 and 0.002, respectively) or in combination (*p* = 0.0001). Subject to the considerations outlined in the discussion this indicated that the injected homogenate from the stomach wall of PD patient A had stimulated the aggregation and deposition of human αSyn and the development of localized SDC/DN pathology in 50% of TgM83^+/−^ recipient mice.

Neurological disease with movement impairment was observed only in one PStH-challenged animal (ID: 60410; Supplementary Table 1, online resource) not showing SDC/DN pathology, and in no animals that had been injected with PMuH or PBld. Thus, no higher incidence or faster onset of neurological symptoms could be detected in TgM83^+/−^ test mice exposed to PStH, PMuH or PlBd than in the negative control animals sham-challenged with human NBH.

## Discussion

### Challenge of TgM83^+/−^ mice with brain tissue from PD patients

In our study, we found that i.c. injected brain homogenates from two PD patients resulted in the consistent detection of a subtle phenotype of cerebral αSyn deposition, referred to by us as localized SDC/DN pathology, in TgM83^+/−^ mice. 54 out of 56 test mice challenged with PBH and eligible to IHC and statistical data analysis showed this pathological phenotype, but none of the 33 corresponding control mice injected with NBH.

#### Did localized SDC/DN pathology represent deposits of residual inoculum?

It can be concluded for three reasons that the localized SDC/DN pathology detected specifically in our test mice did not represent residual exogenous αSyn^PD^ of the inoculated PBH but pathologically phosphorylated and aggregated endogenous αSyn. First, Recasens et al. [31] have shown that exogenously inoculated LB-linked αSyn^PD^ could be detected by 24 h after i.c. injection in the brain of wild-type mice, but not at any later time point between 4 weeks and 17 months post-injection.

Second, homogenized caudate nucleus tissue from PD patients had been injected into TgM83^+/−^ mice. As outlined above, this homogenate contained predominantly presynaptic αSyn^PD^ micro-aggregates instead of LBs, which are only phosphorylated at Ser129 to a limited extent. Our caudate nucleus inoculum showed only a weak immunolabelling of αSyn^PD^ aggregates by anti-pSer129 αSyn antibody ab51253 in the Western blot (Fig. 1b—lanes 2 and 3). The αSyn deposits of localized SDC/DN pathology, in contrast, were immunohistochemically intensely labeled with this antibody, indicating that they consisted of highly phosphorylated, i.e., endogenous αSyn (Fig. 4i–l).

Third, localized SDC/DN pathology could not be detected by us in 4 out of 7 369- to 531-day-old test mice challenged with PBH that were neuropathologically examined at 314–488 dpi, while it was consistently present in 51 test mice with incubation times of > 488 dpi and aged > 531 days. If the localized SDC/DN phenotype would have been based on equally present residual αSyn^PD^ from the inoculum in younger and older animals, this phenotype should have been detected in both groups with no significantly different frequency. However, according to Fisher’s exact test, localized SDC/DN pathology occurred with statistical significance less frequently in the group of younger than in the group of older animals (3 positive out of 7 examined younger mice vs 51 positive out of 51 examined older mice; *p* = 0.0001). This strongly argues against residual exogenous αSyn^PD^ as the molecular species constituting the localized SDN/DN pathology observed in younger and older mice.

Rather, the occurrence of this pathology correlated plausibly with the incubation time and age of our test mice. With the seeding doses injected (≈ 2 × 10^7^ SD_50_ per animal), the earliest occurrence of localized SDC/DN pathology was detected in one animal at 404 dpi (aged 455 days). The incubation period between 404 and 488 dpi (455–531 days of age) then apparently represented a transitional phase in which three animals were found positive for this pathological phenotype, while four other animals were still negative. After this transition phase, i.e., when the incubation period exceeded 488 days (age > 531 days), all eligible test animals had developed localized SDC/DN pathology.

#### Did localized SDC/DN pathology represent an accelerated stage of congenital pathogenesis or a phenotype of cerebral αSyn deposition not intrinsically expressed in TGM83^+/−^ mice?

When the localized SDC/DN pathology detected in our test mice resulted from the deposition of pathologically phosphorylated and aggregated endogenous αSyn this raises the question of how to further characterize that pathology stimulated by the injected inoculum. Did the localized SCD/DN pathology represent a merely expedited precursor of the innate extensive phenotype of SDC/DN pathology typical for TgM83^+/−^ mice, or a new form of cerebral αSyn pathology that would not have occurred at all in the test animals without the action of the injected αSyn^PD^ seeds? In the former case, the administered PBH would have had the effect of accelerating the development of a genetically predisposed αSyn pathology, whereas in the latter case, it would have triggered a novel form of cerebral αSyn aggregation and deposition not innately expressed in TgM83^+/−^ mice.

Noticeably, the distinct phenotype of an apparently stable localized SDC/DN pathology over time observed in our bioassay has not been explicitly described for TgM83 mice so far, either in the context of their congenital characteristics [16] or that of transmission studies [29, 32, 39]. If the localized SDC/DN pathology were a pathogenetic precursor on the innate pathway ultimately leading, without or with acceleration by proteinaceous seeds, to extensive SDC/DN pathology that localized phenotype would presumably have distinctively occurred in these studies.

Moreover, the localized phenotype of SDC/DN pathology should then also have plausibly occurred in our experiments, at least occasionally, in the group of sham-challenged control mice, since 2 out of 35 of these mice had already developed the extensive phenotype of SDC/DN pathology. However, none of the remaining 33 sham-challenged control mice did show signs of localized SDC/DN pathology. Furthermore, if localized SDC/DN pathology were a pathogenetic precursor for extensive SDC/DN pathology, the extensive phenotype would appear likely to occur at a significantly higher frequency in groups of mice with a larger than a smaller proportion of animals having developed the localized phenotype. Yet, although 54 out of 58 mice challenged with PBH displayed localized SDC/DN pathology, only 2 (i.e., not more than in the sham-challenged control group) showed the extensive phenotype of SDC/DN pathology.

Thus, the available body of data is rather suggestive of the localized SDC/DN pathology not being a merely accelerated stage of congenital pathogenesis but a novel phenotype of cerebral αSyn deposition that is not intrinsically expressed in TGM83^+/−^ mice. However, further studies are necessary to definitely resolve this question.

#### Status quo of bioassay findings on the experimental transmission of αSyn^PD^ seeds

The results of our study reconcile the apparently contradictory findings reported by Recasens et al. [31] and Prusiner et al. [29]. At first, our observations are in agreement with Recasens et al. [31] who experimentally induced cerebral αSyn pathology such as diffuse αSyn deposition in the cytoplasm of neurons or increased immunolabelling in synaptic terminals in wild-type mice and rhesus monkeys, respectively, by i.c. injection of Lewy body extracts prepared from human PD brain tissue. However, while Recasens et al. [31] focused on the conversion of murine and simian αSyn, our study did so on that of authentic, albeit A53T-mutated, human αSyn. This allowed the detection of PD-typical pathological characteristics such as pSer129 αSyn positive DNs not displayed in bioassay animals of the study by Recasens et al. [31]. Furthermore, these authors used Lewy body extracts enriched for αSyn^PD^ aggregates as inoculum in their transmission studies, while we injected non-concentrated brain tissue homogenate into our test animal which reflects a more realistic worst-case scenario for iatrogenic αSyn^PD^ transmission.

While our study supported the positive test results by Recasens et al. [31] in this way, it also confirmed the negative findings reported by Prusiner et al. [29] who reported that i.c. inoculation of brain tissue homogenates from PD patients did not result in detectable cerebral αSyn deposition in the same TgM83^+/−^ model as we used. The tissue homogenates tested in these experiments originated from the frontal cortex or the substantia nigra. These specimens were not biochemically enriched or concentrated for αSyn^PD^ which may provide one explanation for the apparent discrepancy to the findings by Recasens et al. [31]. More importantly, we observed that in TgM83^+/−^ mice incubation times of at least about 400 dpi are required to detect stimulation of αSyn deposition in the brain upon an i.c. challenge with caudate nucleus homogenates.

In the study by Prusiner et al. [29], TgM83^+/−^ mice challenged with tissue homogenates from the substantia nigra or frontal cortex were kept for “> 360” dpi until neuropathological examination. Thus, there was a high likelihood of negative test results such as the reported ones if the incubation time (the upper limit of which was not specified in the report) had not substantially exceeded 400 dpi in this study.

However, our study demonstrated that with sufficiently long incubation times, and inoculums containing high levels of presynaptic αSyn^PD^ micro-aggregates and αSyn^PD^ seeding activity, the TgM83^+/−^ mouse model exhibited a clear stimulation of the cerebral aggregation and deposition of endogenous αSyn by injected αSyn^PD^ seeds, and thus provided results consistent with those reported by Recasens et al. [31].

Finally, Prusiner et al. [29] could not detect a stimulation of the occurrence of neurological disease in their PBH-challenged TgM83^+/−^ mice after “> 360” dpi, which is in agreement with our failure to do so up to 612 dpi.

### Challenge of TgM83^+/−^ mice with blood, muscle or stomach wall tissue of a PD patient

While i.c. injection of whole blood or homogenized muscle tissue (biceps) from PD patient A failed to stimulate cerebral αSyn aggregation and deposition in our TgM83^+/−^ test mice, a challenge with homogenized stomach wall from this donor did not so and resulted, with statistical significance (*p* = 0.0001), in the occurrence of localized SDC/DN pathology in 50% of the recipient mice. To the best of our knowledge, this is the first report on a stimulation of pathological αSyn aggregation and deposition in the brain of bioassay animals by transmitted non-CNS tissue from a PD patient.

However, special care must be taken when interpreting this finding, as Sacino et al. [32] reported that a component of normal spinal cord homogenates (NSpH) from naïve non-transgenic mice induced robust αSyn pathology and even severe neurological disease in TgM83^+/−^ mice. Apparently, a component of NSpH other than αSyn seeds did unspecifically, yet fulminantly accelerate the congenital pathogenesis of the animals in these experiments.

Unfortunately, no negative control of non-PD stomach wall tissue was included in our TgM83^+/−^ bioassay, because NBH was considered as sufficient a negative control material by us and the authority for animal protection before the report by Sacino et al. [32] appeared subsequently to the start of our experiments. However, for the following reasons, the stimulation of localized SDC/DN pathology in our animals by injected PStH does not seem to have resulted from an unspecific effect as such caused by NSpH in the study by Sacino et al. [32]:

First, the authors concluded that the NSpH component responsible for the observed unspecific effect originated likely from the white matter, since they did not find an induction of αSyn deposition by injected cortical brain homogenate from normal donor mice. The stomach wall homogenate used in our study, though, did not contain high proportions of white matter material.

Second, TgM83^+/−^ mice injected with NSpH developed an extensive SDC/DN pathology and severe motor disease after very short incubation periods of 112–154 dpi. Our test mice, in contrast, displayed a much milder localized SDC/DN pathology, and no onset of clinical disease, even only after much longer incubation times of 524–566 dpi. Thus, the neuropathological and clinical effects of injected NSpH and PStH differed profoundly from each other.

Third, only 50% of our test mice showed localized SDC/DN pathology whereas all of the NSpH-challenged animals detailed in the study of Sacino et al. [32] developed extensive SDC/DN pathology. This shows that a non-specific sweeping stimulation of extensive SDC/DN pathology as after NSpH injection did not occur in our test mice, again arguing against such effect as cause for the detected phenotype of αSyn deposition.

Fourth, the only localized SDC/DN pathology and negative clinical readout of our PStH-injected test mice corresponded exactly to the findings after a challenge with PBH, with the only difference that the administration of PStH resulted in a lower attack rate of αSyn^PD^ seeding than an injection of PBH.

Fifth, this set of observations can be explained offhandedly by the transmission of αSyn^PD^ seeds via the injected PStH, since our RT-QuIC pilot analysis indicated that this tissue contained seeding-active αSyn^PD^, though apparently at a significantly lower concentration than PBH as demonstrated by the negative Western blot finding for PStH.

In contrast, the alternative hypothesis, according to which the observed seeding effect may be due to an unspecific non-αSyn component in the injected PStH, seems very unlikely in the light of the detection of αSyn^PD^ seeding activity in this tissue. Such alternative hypothesis would require, in a rather implausible way, two premises: (i) that the transferred αSyn^PD^ seeds in the injected PStH did not stimulate the localized SDC/DN pathology, and (ii) that instead an unspecific non-αSyn component in PStH mimicked the pathogenic effect exactly to be expected for the detected αSyn^PD^ seeding activity.

Notwithstanding this, we cannot rule out with absolute certainty that the observed stimulation of localized SDC/DN pathology was caused by an unspecific non-αSyn component of the stomach wall analogously to that reported by Sacino et al. [32] in normal spinal cord homogenates. Yet, based on the findings presented and the considerations made we feel that the cerebral αSyn aggregation in our test mice challenged with PStH can in all probability be attributed to a specific seeding effect of injected αSyn^PD^.

### Conclusions for infection research and clinical practice

I.c. injection of homogenized human PD brain or stomach wall tissue, but not of blood or homogenized muscle (biceps) tissue, from PD patients stimulated cerebral deposition of endogenous pathologically aggregated and phosphorylated αSyn in form of localized SDC/DN pathology in our TgM83^+/−^ mice. In contrast to this clear neuropathological effect of the seeding-active PBH and PStH inocula, these did not induce detectable health impairments in the used animal model. That is particularly noticeable since TgM83^+/−^ mice develop a severe neurological phenotype upon challenge with αSyn seeds from diseased TgM83^+/+^ mice or patients with MSA [29, 39]. At the same time, there has been no epidemiological evidence reported so far for a transmission between humans of severe or even fatal disease by seeding-active αSyn^PD^ aggregates. This has been also recently reviewed by Asher et al. [2] who concluded that there is currently insufficient evidence to suggest more than a negligible risk, if any, of a direct infectious etiology for human neurodegenerative protein aggregation disorders such as PD.

However, for various reasons, it may be difficult to epidemiologically track down any transmissible origin if such cause underlay a subgroup of PD cases. Furthermore, a stimulation of cerebral protein aggregation in humans by iatrogenically transmitted αSyn^PD^ seeds could possibly have harmful effects below full-blown PD transmission [7]. Therefore, possible infectiological risks potentially associated with the transfer of αSyn^PD^ seeds between humans should be carefully considered and further investigated. The same holds true with respect to precautionary measures for patient safety, which in the light of our findings should pay special attention to tissues from the brain as well as from the alimentary tract, the enteric nervous system of which is known to often contain pathological αSyn aggregates in PD (reviewed in: [10, 27]). We did not detect a transfer of αSyn^PD^ seeding activity via blood or homogenized muscle tissue from the biceps, but since absence of evidence does not provide evidence of absence, further studies are necessary to validate this finding.

Currently, there is no experimental or epidemiological justification for new special recommendations modifying existing guidelines on the reprocessing of medical devices such as gastroscopes, colonoscopes or surgical instruments, but potential contaminations with αSyn^PD^ seeds should be removed or inactivated as far as possible when reprocessing these and other medical devices. To achieve this requirement as optimally as possible, the efficacy of physical and chemical reprocessing procedures against αSyn^PD^ seeds needs to be quantitatively assessed on a broader scale. A systematic expansion of the data basis on both transfer risks and precautionary strategies can significantly contribute to adequately counter in the clinical practice the phenomenon of transmissible αSyn^PD^ seeding activity.

## Supplementary Information

Below is the link to the electronic supplementary material.

Supplementary file1 (DOCX 46 KB)

Supplementary file2 (TIF 9201 KB)

Supplementary file3 (DOCX 13 KB)

## Abbreviations

AD: Alzheimer’s disease
Aß: Amyloid-beta protein
BH: Brain homogenate
CNS: Central nervous system
DLB: Dementia with Lewy bodies
DN(s): Dystrophic neurite(s)
dpi: Days post-injection
h: Hour(s)
hMW: High molecular weight
i.c.: Intracerebral
ID: Identity number
LB: Lewy body
min.: Minute/s
MSA: Multiple system atrophy
n: Number
NBH: Non-PD brain homogenate
NSpH: Non-PD spinal cord homogenate
NStH: Non-PD stomach wall homogenate
PBH: PD brain homogenate
PBH A, PBH B: PD brain homogenate from PD patient A or B, respectively
PBld: Whole blood from PD patient
PMuH: Muscle homogenate from PD patient
PBS: Phosphate-buffered saline
PD: Parkinson’s disease
PET blot: Paraffin-embedded tissue blot
PK: Proteinase K
pSer129 αSyn: Alpha-synuclein phosphorylated at serine 129
PStH: Stomach wall homogenate from PD patient
rpm: Revolutions per minute
RT: Room temperature
RT-QuIC assay: Real-time quaking-induced conversion assay
SDC: Somatodendritic compartment
sec.: Second/s
SD_50_: 50% Seeding dose
TBS: Tris-buffered saline
TBST: Tris-buffered saline containing Tween
wt: Wild-type
αSyn: Alpha-synuclein
αSyn^PD^: PD-associated αSyn
