# Correction to: Transmissible α‑synuclein seeding activity in brain and stomach of patients with Parkinson’s disease

**DOI:** 10.1007/s00401-021-02321-3

**Published:** 2021-05-06

**Authors:** Achim Thomzig, Katja Wagenführ, Phillip Pinder, Marion Joncic, Walter J. Schulz-Schaeffer, Michael Beekes

**Affiliations:** 1 Prion and Prionoid Research Unit, ZBS 6—Proteomics and Spectroscopy, ZBS—Centre for Biological Threats and Special Pathogens, Robert Koch Institute, Nordufer 20, 13353 Berlin, Germany; 2 Present Address: State Office for Health and Social Affairs (LAGeSo), Berlin, Germany; 3 Institute of Neuropathology, Faculty of Medicine, Saarland University, Homburg, Germany

## Correction to: Acta Neuropathologica 10.1007/s00401-021-02312-4

In the original publication, Table 2 contains inadvertent inconsistencies with respect to the bank vole bioassay raw data reported in Supplementary Table 1 (Online Resource), and the findings on localized SDC/DN pathology reported for PStH A erroneously represented the ratio of animals with and without localized SDC/DN pathology, instead of correctly the number of animals with localized SDC/DN pathology per number of examined animals. Data provided in the 4th, 8th, 11th and 12th row of Table 2 therefore need to be duly corrected as follows.

Fourth row (male, PStH A): median age—599 (instead of 595); mean age—595 (instead of 599); localized SDC/DN pathology—3/7 (instead of 3/4).

Eighth row (female, PStH A): median incubation period—546 (instead of 541); mean incubation period—541 (instead of 546); median age—599 (instead of 595); mean age—595 (instead of 599); localized SDC/DN pathology—4/7 (instead of 4/3).

Eleventh row (male and female, PBH B): mean incubation period—554 (instead of 550); mean age—606 (instead of 602).

Twelfth row (male and female, PStH A): median age—599 (instead of 595); mean age—595 (instead of 599); localized SDC/DN pathology—7/14 (instead of 7/7).

In addition, the sentence “Of these animals, four females developed localized SDC/DN pathology of sub-phenotype L+ (Fig. 6c)” in the second-last paragraph of the results section should correctly read “Of these animals, three females and one male developed localized SDC/DN pathology of sub-phenotype L+ (Fig. 6c)”.

These corrections do not affect any material findings, claims or conclusions of the study.

