# Supplementary material for: Transmissible α-synuclein seeding activity in brain and stomach of patients with Parkinson’s disease
Source: Acta Neuropathol. 2021 Apr 24;141(6):861–79. doi: 10.1007/s00401-021-02312-4 (PMC8068459; doi:10.1007/s00401-021-02312-4)
Supplement: Supplementary file 1 — Supplementary file1 (DOCX 46 KB) [file 401_2021_2312_MOESM1_ESM.docx]

**Supplementary table 1, online resource
Animals and neuropathological readouts of TgM83^+/-^ bioassay for detection of transmissible αSyn^PD^ seeding activity in PD tissue samples**

|  |  |  |  |  |  |  |  |  |  |  |  |  |  |  |  |  |  |  |  |  |  |  |  |  |  |  |  |  |  |  |  |  |
| --- | --- | --- | --- | --- | --- | --- | --- | --- | --- | --- | --- | --- | --- | --- | --- | --- | --- | --- | --- | --- | --- | --- | --- | --- | --- | --- | --- | --- | --- | --- | --- | --- |
|  |  |  |  |  |  |  |  |  |  |  |  |  |  |  |  |  |  |  |  |  |  |  |  |  |  |  |  |  |  |  |  |  |
| **Inoculum** |  | **Mice** |  |  |  |  |  |  |  |  |  |  |  |  |  |  |  |  |  |  |  |  |  |  |  |  |  |  |  |  |  |  |
|  |  |  |  |  |  |  |  |  |  |  |  |  |  |  |  |  |  |  |  |  |  |  |  |  |  |  |  |  |  |  |  |  |
|  |  |  |  |  |  |  |  |  |  |  |  |  |  |  |  |  |  |  |  |  |  |  |  |  |  |  |  |  |  |  |  |  |
|  |  | **Males** |  |  |  |  |  |  |  | **Females** |  |  |  |  |  |  |  |  |  |  |  |  |  |  |  |  |  |  |  |  |  |  |
|  |  |  |  |  |  |  |  |  |  |  |  |  |  |  |  |  |  |  |  |  |  |  |  |  |  |  |  |  |  |  |  |  |
|  |  |  |  |  |  |  |  |  |  |  |  |  |  |  |  |  |  |  |  |  |  |  |  |  |  |  |  |  |  |  |  |  |
|  |  | **ID** |  | **Incubation period [dpi]** |  | **Age [d]** |  | **SDC/DN  pathology** |  | **ID** |  | **Incubation period**  **[dpi]** |  | **Age [d]** |  | **SDC/DN  pathology** |  |  |  |  |  |  |  |  |  |  |  |  |  |  |  |  |
|  | | | | | | | | | | | | | | | | |  |  |  |  |  |  |  |  |  |  |  |  |  |  |  |  |
|  |  |  |  |  |  |  |  |  |  |  |  |  |  |  |  |  |  |  |  |  |  |  |  |  |  |  |  |  |  |  |  |  |
| **NBH** |  | **4922^a^** |  | **236** |  | **287** |  | **-** |  | **6793** |  | **460** |  | **513** |  | **-** |  |  |  |  |  |  |  |  |  |  |  |  |  |  |  |  |
|  |  | **5631^a^** |  | **378** |  | **429** |  | **-** |  | **5464** |  | **474** |  | **526** |  | **-** |  |  |  |  |  |  |  |  |  |  |  |  |  |  |  |  |
|  |  | **5561^a^** |  | **381** |  | **432** |  | **-** |  | **3850** |  | **535** |  | **586** |  | **-** |  |  |  |  |  |  |  |  |  |  |  |  |  |  |  |  |
|  |  | **0389^a^** |  | **382** |  | **433** |  | **-** |  | **4322** |  | **537** |  | **590** |  | **-** |  |  |  |  |  |  |  |  |  |  |  |  |  |  |  |  |
|  |  | **0078^a^** |  | **385** |  | **436** |  | **-** |  | **3962** |  | **567** |  | **620** |  | **-** |  |  |  |  |  |  |  |  |  |  |  |  |  |  |  |  |
|  |  | **8856** |  | **456** |  | **511** |  | **-** |  | **5632** |  | **567** |  | **620** |  | **-** |  |  |  |  |  |  |  |  |  |  |  |  |  |  |  |  |
|  |  | **1540** |  | **471** |  | **522** |  | **-** |  | **6388** |  | **567** |  | **620** |  | **-** |  |  |  |  |  |  |  |  |  |  |  |  |  |  |  |  |
|  |  | **2230** |  | **505** |  | **553** |  | **-** |  | **7935** |  | **567** |  | **620** |  | **-** |  |  |  |  |  |  |  |  |  |  |  |  |  |  |  |  |
|  |  | **8011^b^** |  | **516** |  | **566** |  | **E** |  | **7936** |  | **567** |  | **620** |  | **-** |  |  |  |  |  |  |  |  |  |  |  |  |  |  |  |  |
|  |  | **1598** |  | **523** |  | **566** |  | **-** |  | **0557** |  | **570** |  | **621** |  | **-** |  |  |  |  |  |  |  |  |  |  |  |  |  |  |  |  |
|  |  | **9066^b^** |  | **531** |  | **581** |  | **E** |  | **1633** |  | **570** |  | **621** |  | **-** |  |  |  |  |  |  |  |  |  |  |  |  |  |  |  |  |
|  |  | **0228** |  | **570** |  | **621** |  | **-** |  | **4664** |  | **570** |  | **621** |  | **-** |  |  |  |  |  |  |  |  |  |  |  |  |  |  |  |  |
|  |  | **0497** |  | **570** |  | **621** |  | **-** |  | **7582** |  | **570** |  | **621** |  | **-** |  |  |  |  |  |  |  |  |  |  |  |  |  |  |  |  |
|  |  | **1081** |  | **572** |  | **622** |  | **-** |  | **7900** |  | **570** |  | **621** |  | **-** |  |  |  |  |  |  |  |  |  |  |  |  |  |  |  |  |
|  |  | **2914** |  | **572** |  | **623** |  | **-** |  | **5443** |  | **571** |  | **625** |  | **-** |  |  |  |  |  |  |  |  |  |  |  |  |  |  |  |  |
|  |  | **6394** |  | **572** |  | **622** |  | **-** |  | **7074** |  | **571** |  | **623** |  | **-** |  |  |  |  |  |  |  |  |  |  |  |  |  |  |  |  |
|  |  | **8816** |  | **572** |  | **623** |  | **-** |  | **5499** |  | **598** |  | **641** |  | **-** |  |  |  |  |  |  |  |  |  |  |  |  |  |  |  |  |
|  |  | **2776** |  | **573** |  | **615** |  | **-** |  | **4849** |  | **612** |  | **654** |  | **-** |  |  |  |  |  |  |  |  |  |  |  |  |  |  |  |  |
|  |  | **5694** |  | **612** |  | **654** |  | **-** |  | **8274** |  | **612** |  | **654** |  | **-** |  |  |  |  |  |  |  |  |  |  |  |  |  |  |  |  |
|  |  | **6875** |  | **612** |  | **654** |  | **-** |  | **9566** |  | **612** |  | **654** |  | **-** |  |  |  |  |  |  |  |  |  |  |  |  |  |  |  |  |
|  |  |  |  |  |  |  |  |  |  |  |  |  |  |  |  |  |  |  |  |  |  |  |  |  |  |  |  |  |  |  |  |  |

**Supplementary table 1, online resource (continued)**

|  |  |  |  |  |  |  |  |  |  |  |  |  |  |  |  |  |
| --- | --- | --- | --- | --- | --- | --- | --- | --- | --- | --- | --- | --- | --- | --- | --- | --- |
|  |  |  |  |  |  |  |  |  |  |  |  |  |  |  |  |  |
| **PBH A** |  | **5242** |  | **505** |  | **554** |  | **L++** |  | **7125** |  | **404** |  | **455** |  | **L+** |
|  |  | **3230** |  | **569** |  | **620** |  | **L+** |  | **4171** |  | **412** |  | **455** |  | **L+** |
|  |  | **7986** |  | **569** |  | **620** |  | **L++** |  | **7469** |  | **510** |  | **558** |  | **L++** |
|  |  | **6717** |  | **572** |  | **621** |  | **L++** |  | **5723** |  | **516** |  | **568** |  | **L++** |
|  |  | **7818** |  | **572** |  | **621** |  | **L++** |  | **4848** |  | **523** |  | **566** |  | **L++** |
|  |  | **8109** |  | **572** |  | **621** |  | **L++** |  | **3529** |  | **540** |  | **590** |  | **L++** |
|  |  | **4851** |  | **580** |  | **627** |  | **L++** |  | **9030** |  | **568** |  | **616** |  | **L++** |
|  |  | **5319** |  | **580** |  | **629** |  | **L++** |  | **1629** |  | **572** |  | **622** |  | **L++** |
|  |  | **6110** |  | **580** |  | **627** |  | **L++** |  | **2727** |  | **572** |  | **620** |  | **L++** |
|  |  | **7792** |  | **580** |  | **627** |  | **L++** |  | **4310** |  | **572** |  | **622** |  | **L++** |
|  |  | **6390** |  | **610** |  | **658** |  | **L++** |  | **6229** |  | **572** |  | **624** |  | **L++** |
|  |  | **7896** |  | **610** |  | **659** |  | **L+** |  | **6445** |  | **572** |  | **624** |  | **L++** |
|  |  | **2075** |  | **612** |  | **655** |  | **L+** |  | **2235** |  | **612** |  | **655** |  | **L+** |
|  |  | **4978** |  | **612** |  | **655** |  | **L++** |  | **6559** |  | **612** |  | **655** |  | **L++** |
|  |  | **6488** |  | **612** |  | **655** |  | **L++** |  | **8579** |  | **612** |  | **655** |  | **L++** |
|  |  |  |  |  |  |  |  |  |  |  |  |  |  |  |  |  |
| **PBH B** |  | **3617^a^** |  | **314** |  | **369** |  | **-** |  | **9031** |  | **488** |  | **531** |  | **-** |
|  |  | **4491^a^** |  | **364** |  | **414** |  | **-** |  | **1982** |  | **512** |  | **568** |  | **L++** |
|  |  | **1976** |  | **449** |  | **504** |  | **L+** |  | **6993** |  | **528** |  | **584** |  | **L++** |
|  |  | **3166** |  | **456** |  | **511** |  | **-** |  | **2037** |  | **553** |  | **608** |  | **L++** |
|  |  | **3154^b^** |  | **469** |  | **522** |  | **E** |  | **4043** |  | **553** |  | **609** |  | **L++** |
|  |  | **9602** |  | **536** |  | **579** |  | **L+** |  | **2492** |  | **570** |  | **623** |  | **L++** |
|  |  | **9907^b^** |  | **559** |  | **602** |  | **E** |  | **3201** |  | **570** |  | **620** |  | **L++** |
|  |  | **6441** |  | **569** |  | **620** |  | **L+** |  | **3651** |  | **570** |  | **620** |  | **L++** |
|  |  | **7872** |  | **570** |  | **620** |  | **L++** |  | **4384** |  | **570** |  | **620** |  | **L+** |
|  |  | **0219** |  | **571** |  | **624** |  | **L++** |  | **7335** |  | **570** |  | **620** |  | **L++** |
|  |  | **0473** |  | **571** |  | **623** |  | **L++** |  | **8826** |  | **570** |  | **620** |  | **L+** |
|  |  | **0763** |  | **571** |  | **623** |  | **L++** |  | **3857** |  | **572** |  | **627** |  | **L++** |
|  |  | **1455** |  | **571** |  | **623** |  | **L++** |  | **2054** |  | **581** |  | **637** |  | **L+** |
|  |  | **3030** |  | **571** |  | **625** |  | **L++** |  | **5178** |  | **581** |  | **637** |  | **L++** |
|  |  | **6689** |  | **572** |  | **624** |  | **L+** |  | **8613** |  | **612** |  | **655** |  | **L+** |
|  |  |  |  |  |  |  |  |  |  |  |  |  |  |  |  |  |

**Supplementary table 1, online resource (continued)**

|  |  |  |  |  |  |  |  |  |  |  |  |  |  |  |  |  |
| --- | --- | --- | --- | --- | --- | --- | --- | --- | --- | --- | --- | --- | --- | --- | --- | --- |
|  |  |  |  |  |  |  |  |  |  |  |  |  |  |  |  |  |
| **PStH** |  | **56938** |  | **518** |  | **572** |  | **-** |  | **60410** |  | **518** |  | **572** |  | **-** |
|  |  | **57159** |  | **524** |  | **577** |  | **-** |  | **57290** |  | **524** |  | **577** |  | **L+** |
|  |  | **58542** |  | **524** |  | **577** |  | **L++** |  | **58463** |  | **524** |  | **577** |  | **L++** |
|  |  | **55443** |  | **546** |  | **599** |  | **L++** |  | **54667** |  | **546** |  | **599** |  | **-** |
|  |  | **59393** |  | **546** |  | **600** |  | **L+** |  | **57994** |  | **546** |  | **600** |  | **L+** |
|  |  | **67769** |  | **568** |  | **620** |  | **-** |  | **73260** |  | **566** |  | **620** |  | **-** |
|  |  | **71950** |  | **568** |  | **620** |  | **-** |  | **75523** |  | **566** |  | **620** |  | **L+** |
|  |  |  |  |  |  |  |  |  |  |  |  |  |  |  |  |  |
| **PMuH** |  | **53956** |  | **497** |  | **551** |  | **-** |  | **69357** |  | **471** |  | **498** |  | **-** |
|  |  | **59358** |  | **517** |  | **571** |  | **-** |  | **59941** |  | **517** |  | **571** |  | **-** |
|  |  | **59002** |  | **551** |  | **600** |  | **-** |  | **55507** |  | **517** |  | **571** |  | **-** |
|  |  | **61550** |  | **551** |  | **600** |  | **-** |  | **55194** |  | **517** |  | **572** |  | **-** |
|  |  | **61769** |  | **551** |  | **605** |  | **-** |  | **55896** |  | **517** |  | **572** |  | **-** |
|  |  | **69620** |  | **559** |  | **611** |  | **-** |  | **60917** |  | **539** |  | **593** |  | **-** |
|  |  |  |  |  |  |  |  |  |  |  |  |  |  |  |  |  |
| **PBld** |  | **58900** |  | **518** |  | **571** |  | **-** |  | **59156** |  | **517** |  | **570** |  | **-** |
|  |  | **54480** |  | **518** |  | **571** |  | **-** |  | **60048** |  | **517** |  | **570** |  | **-** |
|  |  | **54499** |  | **518** |  | **571** |  | **-** |  | **55565^b^** |  | **517** |  | **571** |  | **E** |
|  |  | **59880** |  | **518** |  | **571** |  | **-** |  | **61619** |  | **517** |  | **571** |  | **-** |
|  |  | **54141** |  | **518** |  | **571** |  | **-** |  | **56411** |  | **517** |  | **571** |  | **-** |
|  |  | **67570** |  | **560** |  | **613** |  | **-** |  | **65037** |  | **566** |  | **620** |  | **-** |
|  |  | **73314** |  | **568** |  | **620** |  | **-** |  | **71478** |  | **566** |  | **620** |  | **-** |
|  |  |  |  |  |  |  |  |  |  | **57433** |  | **566** |  | **620** |  | **-** |
|  |  |  |  |  |  |  |  |  |  |  |  |  |  |  |  |  |

^a^ Readout of immunohistochemical (IHC) analysis not usable because incubation period < 400 dpi. ^b^ Excluded from statistical analysis for the detection of αSyn^PD^ seeding to avoid confounding effects. d: Days. dpi: Days post injection. DN: Dystrophic neurite. E: Extensive SDC/DN pathology (with pronounced widespread cerebral deposition of pathologically phosphorylated and aggregated αSyn in the somatodendritic compartment and dystrophic neurites). ID: Identification number (last four or five digits of RFID-chip number for brain or peripheral tissue recipients, respectively) L: Localized SDC/DN pathology (with deposition of pathologically phosphorylated and aggregated αSyn in individual [L+] or focally clustered [L++] somatodendritic compartments or dystrophic neurites). NBH: Non-PD brain homogenate. PBH: PD brain homogenate. PBld: Whole blood from PD patient. PD: Parkinson´s disease; PMuH: Muscle homogenate from PD patient. PStH: Stomach wall homogenate from PD patient. SDC: Somatodendritic compartment.
