# Supplementary figures and images for: Transmissible α-synuclein seeding activity in brain and stomach of patients with Parkinson’s disease
Source: Acta Neuropathol. 2021 Apr 24;141(6):861–79. doi: 10.1007/s00401-021-02312-4 (PMC8068459; doi:10.1007/s00401-021-02312-4)

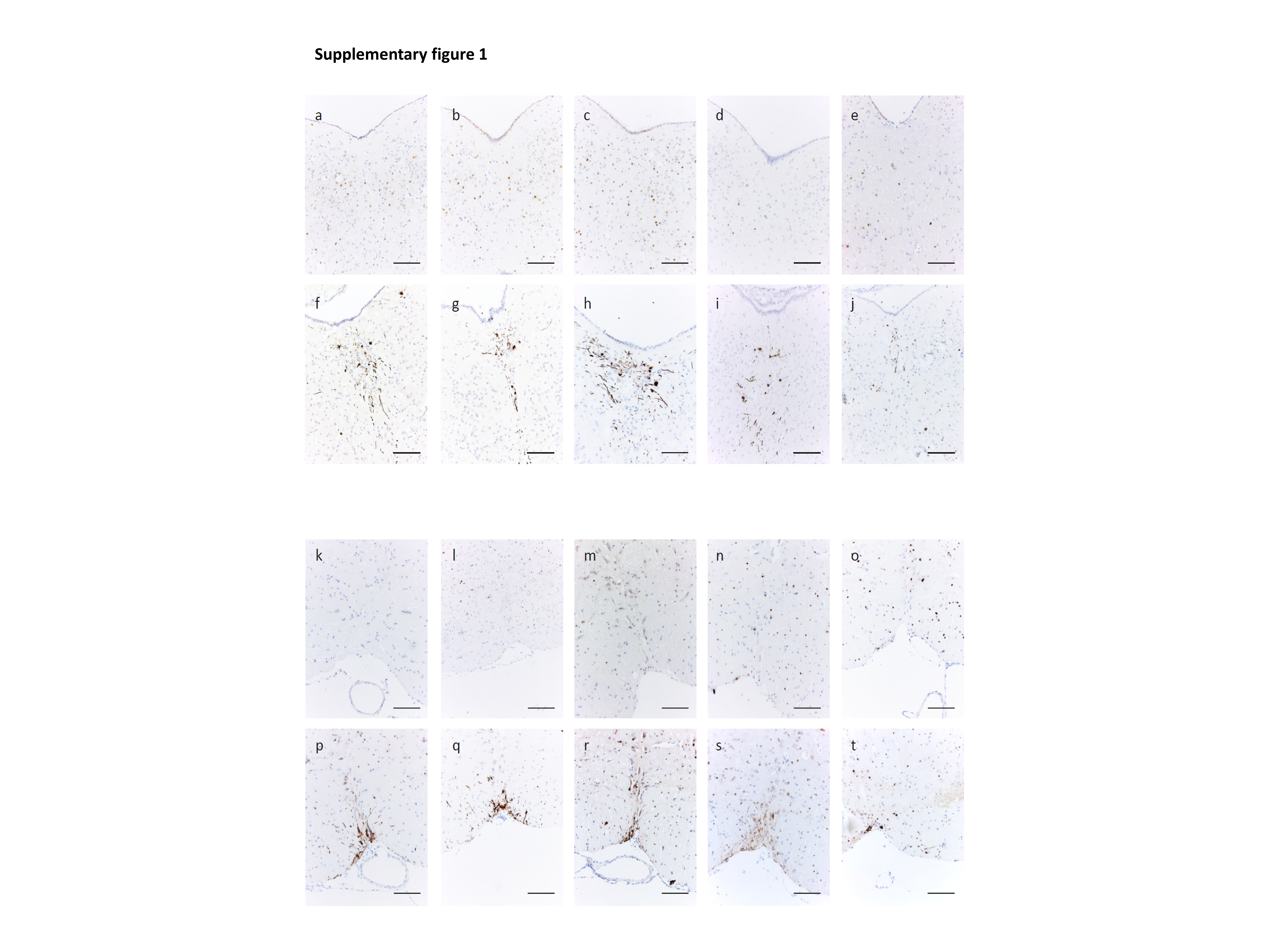

Supplement: Supplementary file 2 — Supplementary file2 (TIF 9201 KB) [file 401_2021_2312_MOESM2_ESM.tif]
