# Supplementary material for: Transmissible α-synuclein seeding activity in brain and stomach of patients with Parkinson’s disease
Source: Acta Neuropathol. 2021 Apr 24;141(6):861–79. doi: 10.1007/s00401-021-02312-4 (PMC8068459; doi:10.1007/s00401-021-02312-4)
Supplement: Supplementary file 3 — Supplementary file3 (DOCX 13 KB) [file 401_2021_2312_MOESM3_ESM.docx]

**Supplementary figure 1** Comparative immunohistochemical analysis of αSyn deposition in the medulla oblongata of TgM83^+/-^ mice challenged with human brain homogenates from a non-PD control donor or PD patients

**a-t** Brain sections from the medulla oblongata of TgM83^+/-^ mice immunohistochemically labelled for phosphorylated human αSyn. Localized (or "L") SDC/DN pathology in the brain of TgM83^+/-^ mice challenged with PBH was easily detectable and strongly contrasted with the lack of such pathology in brain sections of mice sham-challenged with NBH. Absence of localized SDC/DN pathology is shown for the following animals sham-challenged with NBH:
**a** ID 5499 (598 dpi), **b** ID 7074 (571 dpi), **c** ID 4849 (612 dpi), **d** ID 1598 (523 dpi), **e** ID 7936 (567 dpi), **k** ID 4849 (612 dpi), **l** ID 5499 (598 dpi), **m** ID 7582 (570 dpi), **n** ID 2776 (573 dpi), **o** ID 1633 (570 dpi). Localized SDC/DN pathology was most prominent in the area of medulla oblongata in the region of vestibular nuclei at the 4^th^ ventricle (**f-j**) and in the region of pyramidal / reticular nuclei in the same sectional plane (**p-t**). Shown are IHC results from the following TgM83^+/-^ mice challenged with PBH: **f** ID 2037 (553 dpi), **g** ID 2727 (572 dpi), **h** ID 6229 (572 dpi), **i** ID 4043 (553 dpi), **j** ID 8109 (572 dpi), **p** ID 3201 (570 dpi), **q** ID 0763 (571 dpi), **r** ID 4848 (523 dpi), **s** ID 4043 (553 dpi), **t** ID 8613 (612 dpi). Anti-pSer129 αSyn antibody ab51253 was used for immunohistochemical staining. Bars: 100 µm. NBH: Non-PD brain homogenate; PBH: PD brain homogenate; SDC/DN: somatodendritic compartment/ dystrophic neurite.
